# Moving Towards Accountability for Reasonableness – A Systematic Exploration of the Features of Legitimate Healthcare Coverage Decision-Making Processes Using Rare Diseases and Regenerative Therapies as a Case Study

**DOI:** 10.15171/ijhpm.2019.24

**Published:** 2019-05-08

**Authors:** Monika Wagner, Dima Samaha, Roman Casciano, Matthew Brougham, Payam Abrishami, Charles Petrie, Bernard Avouac, Lorenzo Mantovani, Antonio Sarría-Santamera, Paul Kind, Michael Schlander, Michele Tringali

**Affiliations:** ^1^ Analytica Laser, Montreal, QC, Canada.; ^2^ Analytica Laser, London, UK.; ^3^ Analytica Laser, New York City, NY, USA.; ^4^ National Health Care Institute (ZIN), Diemen, The Netherlands.; ^5^ Pfizer Inc, New York City, NY, USA (retired).; ^6^ Liège University, Liège, Belgium.; ^7^ Center for Public Health Research, University of Milan-Bicocca, Milan, Italy.; ^8^ National School of Public Health IMIENS-UNED, Madrid, Spain.; ^9^ Department of Public Health, University of Alcalá, Alcalá de Henares, Spain.; ^10^ University of Leeds, Leeds, UK.; ^11^ Division of Health Economics, German Cancer Research Center (DKFZ), Heidelberg, Germany.; ^12^ University of Heidelberg, Heidelberg, Germany.; ^13^ ASST Niguarda and Regione Lombardia, Welfare Directorate, Milano, Italy.

**Keywords:** Accountability for Reasonableness, Rare Diseases, Multicriteria Decision Analysis, Cost-Effectiveness Analysis

## Abstract

**Background:** The accountability for reasonableness (A4R) framework defines 4 conditions for legitimate healthcare coverage decision processes: Relevance, Publicity, Appeals, and Enforcement. The aim of this study was to reflect on how the diverse features of decision-making processes can be aligned with A4R conditions to guide decision-making towards legitimacy. Rare disease and regenerative therapies (RDRTs) pose special decision-making challenges and offer therefore a useful case study.

**Methods:** Features operationalizing each A4R condition as well as three different approaches to address these features (cost-per-QALY-focused and multicriteria-based) were defined and organized into a matrix. Seven experts explored these features during a panel run under the Chatham House Rule and provided general and RDRT-specific recommendations. Responses were analyzed to identify converging and diverging recommendations.

**Results:** Regarding Relevance, recommendations included supporting deliberation, stakeholder participation and grounding coverage decision criteria in normative and societal objectives. Thirteen of 17 proposed decision criteria were recommended by a majority of panelists. The usefulness of universal cost-effectiveness thresholds to inform allocative efficiency was challenged, particularly in the RDRT context. RDRTs raise specific issues that need to be considered; however, rarity should be viewed in relation to other aspects, such as disease severity and budget impact. Regarding Publicity, panelists recommended transparency about the values underlying a decision and value judgements used in selecting evidence. For Appeals, recommendations included a life-cycle approach with clear provisions for re-evaluations. For Enforcement, external quality reviews of decisions were recommended.

**Conclusion:** Moving coverage decision-making processes towards enhanced legitimacy in general and in the RDRT context involves designing and refining approaches to support participation and deliberation, enhancing transparency, and allowing explicit consideration of multiple decision criteria that reflect normative and societal objectives.

## Introduction

The debate on how best to support priority setting decisions in healthcare has accelerated in recent years due to, in part, a surge in technological innovation (eg, gene therapies), which often focus on previously untreatable, rare diseases (RDs), as well as to economic pressures. A central aspect of the debate is the notion of the legitimacy of the decision. The Oxford dictionary provides 2 definitions of legitimacy^1^: “conformity to the law or to rules” and “ability to be defended with logic or justification,” highlighting both the procedural as well as the substantive aspects of legitimacy.^2,3^ Daniels frames the legitimacy problem as the question under which conditions the moral authority of those who make limit-setting decisions should be accepted as legitimate.^4^ In response, Daniels and his coworkers have developed the accountability for reasonableness (A4R) framework, which defines 4 conditions that can enhance legitimacy and help stakeholders develop a mutual basis for decision-making^5^:

- Relevance (originally termed ‘reasonableness’ condition^6^): As the shared goal of the deliberation is meeting population health needs while taking into account resource limitations, decisions must be based on reasons that can be accepted to be relevant to this goal by all ‘fair-minded’ stakeholders, that is, those who are affected by the decision and who are willing to work together on the basis of reason.^4,6,7^
- Publicity: Requires openness and transparency with regards to the decision itself and the reasons behind it.^4,6-8^ Daniels^7^: “*There must be no secrets where justice is involved, for people should not be expected to accept decisions that affect their well-being unless they are aware of the grounds for those decision*s.”
- Revisability (originally termed ‘appeals condition’^6^): Requires the establishment of mechanisms through which stakeholders can appeal the decision (and it can be revised) on the basis of new evidence or arguments that were originally not duly considered.^4,6,7^
- Enforcement: Refers to enforcement of the other three conditions through voluntary (private) mechanisms or through public regulation.^9^

The A4R approach proposes a procedural framework for making limit-setting decisions in healthcare under resource constrains. It recognizes that stakeholders are likely to agree on a fair process, but may justifiably disagree about the range and relative importance of different values in decision-making.^5^ The lack of guidance regarding the ‘Relevance’ condition, ie, how to ensure that decision criteria are reasonable and relevant to stakeholders, has been one of the major criticisms of the A4R framework.^10-12^ Indeed, the recent debate on how to embed health technology assessment (HTA) into A4R to support coverage decision-making has mainly evolved around ‘Relevance.’^13^ Baltussen et al^14^ stated that to further legitimacy and perceived fairness of decisions, rather than using generic, pre-established decision criteria, the full range of societal values relevant to a particular decision needs to be identified in a process that involves diverse stakeholders.^14^ According to Daniels and colleagues, to be truly relevant and useful to decision-makers, HTA needs to be expanded beyond efficacy, safety and cost-effectiveness to address ethical questions, including, but not limited to, the trade-off between efficiency and equity, which needs to be addressed through a deliberative process.^8,13^

Different approaches to coverage decision-making may have distinct implications for meeting A4R conditions for legitimacy. Some current HTA processes rely on the cost per quality-adjusted life-year (QALY) concept as a guiding principle for resource allocation, with the goal of selecting interventions that will maximize aggregated population health, conceptualized as the sum of QALYs across individuals, for a given level of resources.^15^ The incremental cost-effectiveness ratio (‘cost/QALY’), however, is not the sole basis for decisions; other factors (eg, the innovative nature of the technology^16^) are usually also considered.

The principal advantage of the use of QALYs is that it represents a generic measure of health which can be used across therapeutic areas. However, three principal ethical concerns have been raised with respect to resource allocation^17^: failure to give priority to those who are worst off (in terms of health or social standing); potential for discrimination against patients with disabilities and comorbidities who, when receiving the same intervention, will likely incur a smaller QALY gain than patients not affected by other conditions; and failure to account for qualitative differences in outcomes (eg, life extension vs quality of life improvement). Additionally, it can be debated whose utilities (ie, QALY weights) should be employed, those of patients, health experts or the general public.^17^

Over the last decade, various multicriteria approaches for measuring the value of healthcare interventions and supporting coverage decision-making have also been developed.^18-21^ These involve explicit consideration of multiple decision criteria, defined in relation to the objective of the decision and structured by applying multicriteria methodology.^22^ These approaches have the potential of consistently incorporating a broader range of criteria that stakeholders might find relevant for a given decision problem in line with their individual value systems and perspectives.^23-25^

While implementation of the A4R framework in current practice of health technology coverage decision-making has been examined,^26-30^ a fundamental understanding of the features that would be considered most conducive to a legitimate process should advance the debate and provide guidance for the development of methods, frameworks and approaches best suited to move towards that goal. In particular, approaches to help HTA processes address the tension between the goals of meeting individual patient needs, serving the whole population equitably, and ensuring health system sustainability, need to be attended to and further explored.^31,32^ Striking a balance between these goals can become particularly challenging when appraising treatments for RDs and regenerative therapies (RTs) (such as, for example, orphan medicines and gene or cell therapies) due to high unmet needs, small patient populations and the often complex and high-cost nature of these treatments.^25,33-35^ In addition, assessing these therapies can be challenging due to specific issues in clinical evidence development, including small and often heterogeneous trial populations (with possibly varying disease classifications), lack of disease-modifying comparator therapies, and uncertainty about long-term outcomes.^25,33-37^ For these reasons, RDs and RTs provide a highly relevant case study for developing guidance on legitimate HTA approaches.

Using A4R framework as a reference, the objective of this study was to reflect on how the diverse features of decision-making processes can best be aligned with conditions that promote A4R (Relevance, Publicity, Appeals, and Enforcement) to guide healthcare coverage decision-making towards enhanced legitimacy, in general, and within the specific context of RDs and RTs.

## Methods

HTA thought leaders explored and discussed during a panel session on how best to address the 4 A4R conditions for legitimacy and provided pertinent recommendations, in general, and within the specific context of RDs and RTs. For this purpose, a tool, the Legitimacy Exploration Matrix (LEM), was developed for this study and provided to the panelists to support their reflection and discussion on how to best align with the A4R conditions in coverage decision-making. Panelists’ comments and recommendations were collected, analyzed and synthesized.

### The Legitimacy Exploration Matrix

The LEM was developed to be used as a platform to support reflection and discussion during the panel session. Specific features that operationalize the 4 A4R conditions in decision-making processes were defined and organized in the matrix by the A4R condition to which they pertain. Identification of these features was supported by a review of the literature on the requirements for legitimate and accountable decision-making processes,^4-10,38^ including the A4R framework and its implementation in HTA processes.^8,13,14,26-28,30,39-42^ (Please refer to Supplementary file 1 for search strategies). A total of 34 features for operationalizing the A4R conditions were thus defined: 26 features for the A4R *Relevance* condition (including 19 features related to decision criteria, 3 to evidence and 4 to deliberation), 3 for the *Publicity* condition, 2 for the *Appeal* condition, and 3 for the *Enforcement (*or* Implementation)* condition. (A condensed version of the LEM is available in Tables 1 to 4, the complete LEM is available as Supplementary file 1 – Appendix 1).

**Table 1:** Panel Recommendations With Respect to A4R Condition 1: Relevance

**Feature**	**What Could Be Recommended for This Feature in General?**	**What Could Be Recommended for This Feature With Regard to RDs and RTs Specifically?**
Facilitating participation in the committee of decision-makers that represent diverse perspectives	*What could be recommended to facilitate participation of diverse perspectives (eg, patients, healthcare professionals, administrators, citizens)?* Multicriteria reflective approach best to facilitate participation; cost/QALY and algorithmic MCDA may not be fully applicable in all cases Define form/extent of participation: providing substantive input (ie, empirical evidence), interpreting results, or deliberating as equal partners? Who is entitled to participate? How to guarantee that all participants are able to properly process the technical/scientific information? Prior stakeholder engagement to develop common understanding, clear definition of roles and expectations Direct the committee clearly and reduce charisma issues. Provide good-quality synthetized information before committee meeting Ensure representation and participation also during the process of evidence generation	• In RDs, patients likely to be the best experts in their disease (do not have the same view as clinicians); reflective multicriteria the only way forward if the decision is to be legitimate • Legitimacy requires involvement of all stakeholders, but final decision is with the Ministry of Health
**Features Related to Criteria**		
Criteria selection process	*What could be recommended regarding who should select the criteria?* Political decision (elected governments) Decision-making committee Public deliberation and majority voting in line with constitution and applicable laws Institution with broad consultation (including workshops) of all stakeholders (eg, specialists, patient associations, patients, economists) Establish rules for weighting the importance of criteria (measuring criteria weights) Flexibility (open to re-visit criteria set) Accept the values obtained or construct the values? *What could be recommended regarding overall goal/values from which criteria are derived?* Typically derived from a nation’s constitution Deliberation about the actual goals that underlie decisions Overall population health values; these may be in conflict of the interests of specific groups They should reflect social norms and preferences elicited from the population *Should there be consistency of criteria across decisions?* Consistency is a pre-requisite for accountability and legitimacy and is crucial to insure equity across disease areas Not necessarily; transparency of the criteria and their weights is more important Yes, but we need a general generic framework that is flexible enough to adjust to specific issues	• Aim for in-depth understanding of how the actual decision is rooted in the fundamental goals of healthcare (eg, allow stakeholders to observe other decision processes) • Adaptation to RDs: impact of number of patients and chronicity of the disease on the evaluation • If we design a system for dealing with all interventions (including RDs and RTs), we may create procedures that are inefficient for many of them • Same rules for all technologies: humanistic; clinical, economic criteria • No major difference to common diseases
*Generic Criteria and Rationales (ie, criteria are used across decisions)*			
*Domain: Effect of intervention*		
Comparative effectiveness	*Should the comparative effectiveness of the proposed intervention be considered and why?* - Yes (7/7) Comparative effectiveness is the cornerstone of decisions because it reflects the type and extent of effect for the patient, which is directly rooted in the goal of the healthcare system Need to justify the choice of comparator Problem of defining a common “health gain” measure across diseases	• Assessment of orphan drugs needs adaptation (eg, use of lesser quality data) due to lack of comparators and (in a first step) use of intermediate rather than final outcomes • Requirements for proof of clinical effectiveness should not be relaxed, not even for ultra-rare disorders
Type of benefit (eg, curative treatment, preventive intervention)	*Should the nature of the clinical benefit provided by the proposed intervention (eg, symptom relief, life extension, cure, prevention) be considered and why?* - Yes (4/7, 1 no, 2 not specified) Yes, offers space for value claims; patient perspective may differ by type of benefit Prevents programs from prioritizing only one kind of benefit; need to distinguish between preventive and curative benefits because prevention can have collective benefit Important for coverage decisions; however, difficult or infeasible to rank. Prioritization of quality over quantity of life (or vice versa) should be backed by specific evidence No need to differentiate between types of benefit; effect on outcomes captures this concept	• RD patients are the only judges of their condition • Might prove infeasible for coverage decisions • Type of benefit may not be known at time of decision (eg, gene therapy may possibly cure a disease but needs long-term evaluation)
Safety/tolerability	*Should the safety/tolerability of the proposed intervention in relation to alternatives be considered and why?* - Yes (6/7, 1 no) Only tolerability known at the time of decision, safety data comes only after drug is used in clinical practice Important part of the decision No, safety/tolerability should be established prior to reimbursement decisions	• In RDs with few and small randomized controlled trials patients could accept higher risks • Pharmacovigilance not helpful in RD and RT setting • Nothing specific
Patient-perceived health/PRO	*Should patient-perceived health/PRO generated by the proposed intervention in relation to alternatives be considered and why?* - Yes (7/7) Yes, otherwise only life-extending interventions would be developed Yes, always, even if data is less frequently available. Patients’ assessment of their own outcomes should be an integral element of the evidence for a therapy Advantage of QALY: cross-disease comparability, but disease-specific measures better to capture treatment effect Yes, but apply the same documentation standards as for clinical endpoints	• Individual changes in PRO levels are more important than absolute PRO levels • Inclusion of disease-specific PRO instruments in clinical trials will require their validation, which is, however, not feasible prior to the trial • Nothing specific
*Intervention-specific (eg, disease-specific outcomes)*	*How should intervention-specific criteria be included and why? (eg, outcomes)* Intervention-specific outcomes are crucial to assess efficacy Important to determine disease-specific outcome measures and make them comparable (harmonization), but this may not always be feasible	• RD and RTs are very context-specific; may need specific criteria • Identify information needs for RDs
*Domain: Disease severity and unmet needs*		
Availability of alternatives (unmet needs)	*Are the availability of alternatives and their shortcomings in their safety/tolerability or in their ability to prevent, cure, or improve the targeted health condition or ameliorate patient-perceived health considered and why?* - Yes (4/7, 1 no, 1 not specified, 1 no data) Yes, but what can be seen as ‘alternatives’ needs to be defined Yes, essential for evaluating the benefit of an intervention; reflects justifiable resource allocation Yes, should be assessed as part of the comparative effectiveness domain Considered in practice but not a robust decision criterion	• Comparators are often absent; can be challenging to define “usual care” • Crucial for RDs • Nothing specific
Disease severity	*Should the severity of the targeted health condition with respect to mortality, morbidity, disability, impact on function and quality of life, and clinical course be considered and why?* - Yes (5/7, 1 no, 1 not specified) Yes, raises awareness of the goal of decision-making A major point. There is social consensus that the most severely affected people should be treated first. Overwhelming empirical evidence for a strong public preference, backed up by normative considerations Operationalization of this criterion requires a metric to measure severity. Priority conditions should be defined on a collective level Linked to ‘Type of benefit.’ Disease severity is irrelevant when considering marginal benefits	• RDs are often severe • Nothing specific
*Domain: Economics*		
Cost (price) of intervention	*Should the cost (price) of the proposed intervention (includes acquisition and administration) in relation to current alternatives be considered and why?* - Yes (5/7, 2 not specified) Crucial for healthcare system sustainability and for value-based use of resources The cost itself is essential, not just the incremental cost in relation to alternatives Although price considerations are out of scope of HTA, inclusion of costs in HTAs informs opportunity cost considerations Yes, Consider also that payers and insured taxpayers/health plan members may have different perspectives Budget impact is important	• Remember that costs for “personalized/precision medicine” are paid for by society’s solidary • Expenses for RDs are becoming too large; need new (collaborative) ways of developing treatments • Need to look at costs as a lifetime approach • High cost, but budget impact acceptable from a political point of view • Role of rarity needs to supported by further empirical studies. Depending on the type of intervention and its underlying economics, there could be specific considerations with respect to RDs
Consequences of intervention for other medical costs	*Should the impact of the proposed intervention on other medical costs (apart from interventions that are directly replaced) be considered and why?* - Yes (5/7, 1 not specified, 1 no data) Yes, clarifies the value proposition and opportunity costs; reflects general principles of health economics Although logical to include, problem of “silo” budgeting limits the consideration of other medical costs Less important than direct costs (should be just explored, depending on what these costs are); maybe important for innovative therapies	• Other medical costs often negligible compared to the cost of therapy
Consequences of intervention for non-medical costs	*Should the impact of the proposed intervention on non-medical costs (eg, lost productivity, care giver time, social services, disability costs) be considered and why?* - Yes (4/7, 1 no, 1 not specified, 1 no data) Generally not considered but should be in principal Relevant from the perspective of patients and families Less important than direct costs but can be explored In public-payer systems, probably unreasonable to include costs that are not covered by the public payer	• Should be considered but often negligible compared to treatment costs • Much more important for conditions with high disability
Budget impact, affordability and opportunity costs	*Should the budget impact, affordability and the opportunity cost of the proposed intervention be considered and why?* - Yes (6/7, 1 no data) Affordability is becoming more and more important; opportunity cost always to be considered Yes, budget impact reflects a change in focus from individual patient costs to program costs	• Despite low number of patients, budget impact can be significant • Any RD specificities can be reflected by the program costs approach (ie, budget impact) and social cost value analysis
ICER	*Should the incremental cost-effectiveness ratio of the proposed intervention be considered and why?* - Yes (4/7, 1 no, 2 not specified) ICER must be placed in context of other attributes of the disease/patient/treatment Although necessary to provide, does not afford appropriate information for decision-makers ICER is important to inform and document the decisions No, that would amount to double-counting in an MCDA framework May be used to examine whether added benefits justify added costs when comparing interventions targeting the same condition (ie, productive efficiency). Should not be used to inform allocative efficiency/opportunity cost considerations across disease areas When there is no mortality impact, use cost-effectiveness (eg, cost per event avoided) rather than cost-utility because it has a real-world meaning to decision-makers	• ICER not very informative. Regulators and reimbursement committees treat RDs differently • Serious methodological flaws for decision-making in RDs and RTs • Difficult to apply; may be used to “justify” high price • Never use for RDs
*Domain: Ethical, social and legal aspects*		
Rarity/Size of affected population	*Should the rarity of the condition and/or the size of the population targeted by the proposed intervention be considered and why?* - Rarity: Yes-No (3 yes, 3 no, 1 not specified); Size of population: 1 yes, 6 not specified Yes, rarity is usually considered No, rarity by itself not meaningful without relation to other aspects, such as severity, budget impact, complexity of care Small population requires a different approach to assessment; MCDA more pertinent Rarity is problematic and difficult. Danger that this concept could be misused to justify higher prices No, but consider a separate budget dedicated to RDs Yes, if cost/patient or cost/QALY (rather than budget impact) are used as benchmarks	A designated fund for RD and RT therapies would make this a different kind of decision, but do societies prioritize specific services to specific populations? Rarity should not be used to ask for high price but could suggest distinctive financing (payment) pathways
Prioritized populations	*Should prioritization of specific populations (eg, vulnerable populations), as defined by policy decision-makers/societies, be considered and why?* - Yes-No (2 yes, 1 no, 4 not specified) Depends on history, beliefs and political environment Yes, but priorities should be set and discussed publicly Always considered; better to make this explicit Strictly political question, outside of analysis of costs and benefits Priorities should be captured through formal analysis (ie, social cost value analysis)	• RD patients usually prioritized; severity and age play a crucial role (‘vertical equity’) • RDs and RTs should not compete with other interventions; need a separate evaluation process
Feasibility of implementing intervention	*Should the capacity of the healthcare system to appropriately implement the proposed intervention with respect to infrastructure, organization, skills, legislation requirements etc. be considered and why?* - Yes (6/7, 1 no) Yes, essential but never properly assessed. Need to ensure that potential benefits are realized in clinical practice. This is an input but also an output of the evaluation (recommendation) Define reference centers of excellence for innovative treatments Not as part of the initial evaluation, but important when it comes to implementation	• Very important for RDs: need to recognize implementation hurdles • Ultra-RDs can have very specific procedures; care often delivered at tertiary referral centers
Political, historical and cultural considerations	*Should the political, historical and cultural context be considered and why?* - Yes-No (No 3/7, 2 yes, 1 not specified, 1 no data) No, we need a systematic approach; nevertheless, in practice highly influential and inevitable Can provide important insights and adds value for collective learning in decision-making Should only be considered informally (qualitative approach); maybe a checklist to identify patient barriers	• Issues more pronounced
Innovative-ness	*Should the concept of innovation be considered and why?* - No (5/7, 1 yes, 1 not specified) No, the meaningful aspects of innovation (‘making a difference’) are already covered by other criteria How to define and fund ‘innovation’? How to control its spread? Audit new therapies to see how they perform in practice in order to enhance learning and support best and proven innovations In principle, yes, as it relates to dynamic efficiency	• Innovation is the rule in these therapeutic areas
*Uncertainty of evidence*			
Degree of uncertainty related to evidence (quality of evidence)	*Should the relevance and validity of the evidence supporting the proposed intervention as well as the degree of uncertainty related to this evidence be considered and why?* - Yes (Yes 5/7, 2 not specified) Sophisticated algorithmic methods might not be helpful Real-world evidence contributes little to reducing uncertainty because of problems with validation Uncertainty should be distinguished from quality of evidence Improving ‘uncertainty’ does not necessarily lead to better decisions Yes, assessment should include effect size and degree of confidence	• Uncertainty should be placed in the context of rarity: typically, small samples show large effects but low precision. Accept lower-quality data than usually required • Important to separate uncertainty from the magnitude of the measured effect • Real-world evidence needed to evaluate long-term benefit of RDs and RTs • Higher uncertainty can only be accepted temporarily
**Features Related to Evidence**		
Considering different types of evidence	*What type of evidence should be considered: scientific, colloquial (“anything that that establishes a fact or gives reason for believing something”* ^7^ *) imputed by logic, insights/ experiential?* For some parameters, scientific evidence is needed, for others colloquial, but relevance must be justified randomized controlled trials do not address clinically relevant questions; therefore need expert evidence Any kind of evidence, but hierarchy: scientific, social “science,” expert opinion; keep them separate in assessment but consider jointly in appraisal Apply principles of evidence-based medicine within a pre-specified decision-making structure; allowing anything is likely to obscure rather than inform	• Most important for RDs • For RTs and RDs moving towards more “open concept” of providing evidence
Selection of evidence	*What should drive the selection of evidence to be included in the assessment?* Relevance to the question at hand; relevance needs be justified Scientific robustness is important, but there are also other elements Not only systematic review; allow any stakeholder to suggest evidence	• Include all relevant evidence
Presentation of evidence	*How should the evidence be presented to enhance clarity and support deliberation?* Synthesized evidence and criteria side-by side (“by-criterion report”), key points in Executive Summary. Need to include uncertainty on evidence for each criterion Systematic review style; discuss strengths and weaknesses of evidence base Need transparency about what is known and unknown (data gaps)	• Transparency also about uncertainty
Balancing values at stake	*How should the values at stake be balanced during the deliberation?* Committee composition has to reflect the different values that exist in society Balancing values should remain a discursive task: weighting should inform and structure deliberations not replace them. To ensure legitimacy and transparency, deliberations must be well documented *Who should balance the values at stake?* Decision-makers, typically politicians Diverse stakeholders should deliberate to identify which/whose values are at stake. A compromise must eventually be made, based on well-documented deliberations Stakeholders should bring their own values	• NA
Assessing the performance of the intervention*	*How should the performance of the intervention be assessed?* Empirical testing Based on its real-world impact *Who should be assessing the performance?* HTA units or researchers, but design of assessment must be in advance agreed upon between researchers and the committee Independent appraisal committee with representation from all stakeholder groups	• NA
Including individual interpretations to reach a group equilibrium in formulating a decision	*How should individual interpretations be included/shared to reach a group equilibrium in formulating a decision? (eg, consensus, vote)* If consensus can initially not be reached, give additional time for reflection. Committee members should be convinced that changing their minds is not a sign of weakness but of learning and reflection Voting if consensus cannot be reached	• NA
Decision rules and uncertainty	*Should there be decision rules to guide the decision-making? What should they be?* Decision rules can enhance transparency. If used, they should support and not replace deliberation and decision-making. They need to be well justified Multicriteria approaches will need some sort of a threshold, but rooted in deep reflection on priority setting and the goals of the healthcare system and on opportunity costs *How should uncertainty in decision-making be handled?* Sophisticated tools for quantifying uncertainties (eg, value of information analysis) may be of limited use in actual decision-making	• ICER threshold may prove legally and politically unfeasible

Abbreviations: A4R, accountability for reasonableness; QALY, quality-adjusted life-year; MCDA, multiple criteria decision analysis; RD, rare disease; RT, regenerative therapy; PRO, patient-reported outcomes; ICER, Incremental cost-effectiveness ratio; HTA, health technology assessment. * NOTE: In this context, performance is defined as how good an intervention is in regard to a specific decision criterion (eg, highly efficacious = high performance with regard to efficacy).

**Table 2:** A4R Condition 2: Publicity

**Feature**	**What Could Be Recommended for This Feature in General?**	**What Could Be Recommended for This Feature With Regard to RDs and RTs Specifically?**
Transparency of criteria and evidence considered and approaches used to consider them	*Should the evidence that was considered and the methods to select and synthesize the evidence be made public?* • Yes, always and unconditionally • Yes, should be described adequately and justified meticulously (including all value judgments used in the process of generating and selecting evidence), then made public • *Should the criteria and the deliberative approach to consider them be made public?* • Yes, deliberations and advice published on the website of the HTA agency Protect individuals according to Chatham House Rule	• Additional attention to justifying the selected endpoints
Understandability of reasoning behind decision	*What could be recommended to facilitate making reasons leading to the decision explicit and understandable to stakeholders, including the public?* • Keep it simple and understandable. The art of good documentation and communication needs to be more valued and further developed • Communicate honestly about uncertainties and imperfection of decisions • Include a natural language account of the process, the key evidence used and the facts	• Knowledge brokers and health anthropologists can play an important role. Avoid emotion-laden communications, which are counterproductive for legitimizing decisions
Clarity of values underlying the decision	*Should values underlying decisions be stated? Should there be a reference to the broader objectives and underlying mandate of the agency/institution/healthcare system?* • Yes, generally. Also, the extent to which the intervention contributes to or infringes upon the mandate of the healthcare system and broader societal objectives must become explicit • Clarify the weights of the different criteria in the final decision	• Public health impact is generally low in RDs, we have to address the fact that incidence and prevalence are low

Abbreviations: A4R, accountability for reasonableness; RD, rare disease; RT, regenerative therapy; HTA, health technology assessment.

**Table 3:** A4R Condition 2: Appeal and Revision

**Feature**	**What Could Be Recommended for This Feature in General?**	**What Could Be Recommended for This Feature With Regard to RDs and RTs Specifically?**
Handling of potential disagreements from stakeholders	*What could be recommended to facilitate consultation to collect feedback from stakeholders on interpretation of data, rationale for decision, values considered and/or reduce the need for appeal?* • Inclusion of different stakeholders in multi-lateral deliberation • Make decision-making rules explicit • Transparency and publicity of the debates • Publish minority reports. Sponsor lay stakeholder rebuttal statements	• NA
Handling new evidence or new context	*What could be recommended to facilitate considering new evidence or new context?* • Need a precise definition of ‘new evidence’ • Publish rules regarding appeals and reviews, including fixed review dates. However, allow for fast-track recall/review in case of emergence of pivotal new evidence • Communicate that decisions are provisional and will be updated according to evidence • Consider CED	• CED important for RDs and RTs. This should involve active and constant improvement of the evidence generation framework • Evidence generation and documentation of clinical outcomes are needed. Performance contracts are useful in this situation

Abbreviations: A4R, accountability for reasonableness; RD, rare disease; RT, regenerative therapy; CED, coverage with evidence development.

**Table 4:** A4R Condition 4: Implementation (or Enforcement)

**Feature**	**What Could Be Recommended for This Feature in General?**	**What Could Be Recommended for This Feature With Regard to RDs and RTs Specifically?**
Existence of means to enforce condition 1	*What could be recommended to ensure that all relevant criteria and evidence are considered in a deliberative process that is inclusive of diverse perspectives?* • External and independent quality review of final deliberations by unconflicted third parties • Need to represent different stakeholders and all relevant disciplines (including, ethics, psychology, law, etc) • Conditions for effective deliberation must be created and safeguarded (eg, skilled, independent moderator) to foster learning and prevent defensiveness	• RDs and RTs well suited to pilot stakeholder deliberations
Existence of means to enforce condition 2	*What could be recommended to ensure publicity of the rationales of the decision, ie, that the reasons behind decisions are understandable and the values on which they are based are clear?* • Use of visualization tools to clarify the rationale of the decision • Promote a culture of collective learning among all stakeholders (including industry, payers, clinicians, patient organizations, etc) • Linked to the weights of different domains underlying the decision	• NA
Existence of means to enforce condition 3	*What could be recommended to ensure revisability of the decision in light of new evidence or arguments?* • Need rules for integrating re-evaluation in pricing and reimbursement processes (‘life cycle’ approach) • Development and piloting of CED arrangements • Define objective of the re-evaluation (new clinical outcome or outcome in real life) and evidence requirements to generate ‘relevant’ rather than ‘more’ evidence • Develop HTA methodology: both conceptual and empirical work on multicriteria decision-making • Need to ensure that reasons for a decision are properly documented to allow later re-assessment	• RDs and RTs are important areas for implementing these recommendations

Abbreviations: A4R, accountability for reasonableness; RD, rare disease; RT, regenerative therapy; CED, coverage with evidence development; HTA, health technology assessment.

For each feature, specific questions were developed to further clarify the concept covered and collect targeted recommendations from the panelists. For example, for *Understandability of the reasoning behind decisions* (a feature for operationalizing the Publicity condition), the question was: *What could be recommended to facilitate making explicit the reasons leading to the decision and understandable to stakeholders, including the public?*

Defining features for operationalizing the Relevance condition required defining potential decision criteria. In order to ensure that a wide range of potential criteria would be included to be discussed during the panel session a systematic review was carried out to identify published multicriteria frameworks that have been proposed to be applicable to interventions targeting RDs (see Supplementary file 1 for search strategies- Appendix 2). The rationale for this approach was that these are generally the most comprehensive decision frameworks (up to 20-21 criteria) and include criteria that are proposed to be relevant for the specific context of RDs and RTs. Seven multicriteria frameworks were thus identified^23,25,43-47^ and the decision criteria that each of them proposed extracted and matched in tabular format (see Supplementary file 1 – Appendix 3). From this list of proposed criteria, those were included in the LEM that were featured in at least two of these frameworks, unless the rationale offered for the proposed criterion (by the authors of the respective framework) was based on price justification alone (eg, manufacturing complexity).

To explore during the panel session a range of possibilities by which each feature could be addressed, three general approaches to decision-making (‘archetypes’) were defined in the LEM^5,15,23-25,31,41,48-56^: (1) The classical cost-effectiveness approach, being rooted in a variant of utilitarian thought, strives to maximize aggregated population health using the cost-per-QALY ratio as a pre-established, dominant decision criterion. Under this archetype, the decision-making committee’s deliberation focuses on interpreting the cost-per-QALY model and its output, but may also extend to other potentially relevant factors. (2) The algorithmic multiple criteria decision analysis (MCDA) approach defines criteria specifically for the intervention being appraised to construct an MCDA model. Stakeholders’ preferences are collected across criteria and value functions are constructed (usually by analysts) for measuring the intervention’s performance with respect to each criterion. The committee’s deliberation focuses on interpreting the MCDA model and its outputs and may take other factors into account qualitatively. (3) The reflective multicriteria approach proposes a generic decision criteria set, derived from the goals of health systems (which can be formulated as meeting individual patient needs, serving the whole population equitably, and ensuring financial sustainability^32^) as well as the principle that decision-making should be informed by best knowledge and understanding of the context.^31^ (Additional health system goals may include responsiveness to legitimate expectations of the population as well as fair financing^57^). Committee members’ deliberation involves judging the intervention’s performance on each criterion (qualitatively or quantitatively) and reflecting on the relative importance of the criteria.

For each feature, an option on how it could be addressed was specified within the LEM for each of these archetypes. Some of these options are inherent to an archetype, while others are not; to stimulate discussion during the panel session, the non-inherent options were specified in a way that would differentiate between the approaches represented by the three archetypes.

Specificities related to RDs and RTs pertaining to HTA and coverage decision-making were also identified for each feature (as applicable) (informed by the key literature,^25,35,36,58,59^ see Appendix 2 for search terms) and included in the LEM to support reflection on the responsiveness of coverage decision-making processes to the unique issues raised by RDs and RTs.

### Panel: Recruitment, Session, Data Collection

Panelists were invited to a half-day face-to-face session in Rome, Italy, in June 2017. Panelists were identified and invited based on their experience and expertise in shaping HTA processes and their interest in and contribution to exploring the role of values, ethics and multicriteria approaches in coverage and reimbursement decision-making. Intending to include perspectives from diverse health systems, ten though leaders from 9 countries across Europe, North and South America were invited to participate. Seven of them, 1 each from France, Germany, the Netherlands, Spain, and the United Kingdom, and 2 from Italy, agreed to participate in this study. Note that all 7 panelists are co-authors of this study (PA, BA, PK, LM, ASS, MS, and MT). Their fields of expertise encompassed (among others) medicine, health services research, health economics, health outcomes research and HTA and their current or past roles practicing clinician, journal editor, coverage decision-maker, and HTA process designer and administrator.

Panelists received the panel manual, containing the LEM, prior to the session. During the session, panelists were first presented with the LEM methodology. Then, each feature was presented and discussed in the group, followed by the panelists recording their individual inputs in writing. To encourage openness of discussion and free exchange of ideas, the session was conducted under the Chatham House Rule so that the comments made cannot be attributed to any individual.^60^ No consensus seeking was attempted. The session was recorded. Completed panel manuals were collected immediately following the session (some panelist completed part of their manuals after the session, which were then collected per email).

### Data Analysis

The written responses provided by the seven panelists in the manuals were the primary data sources. In addition, transcripts of the oral comments of the panelists during the session were also reviewed and used to clarify the meaning of the written inputs in order to ensure a correct understanding and representation of the overall flow and emphasis of the discussion. The panelists’ responses were analyzed using a thematic analysis approach (see details in Appendix 4). The features developed in the LEM were instrumental in our thematic data analysis. In a first step, panelists’ responses were organized by the LEM feature to which they pertained in tabular format. In a second step, within each feature, respondents’ accounts that involved convergent ideas or similar recommendations were identified and combined into one theme, in such a way that preserved the key elements of the original wording of the responses. (The 4 tables in the Results section present these synthesized themes for each A4R condition and feature). Responses to questions about whether a specific decision criterion should be considered and why were analyzed in the same manner; in addition, with respect to the closed parts of these questions, these responses were also categorized as positive (Yes), negative (No) or neither of these (Not specified). These were then counted to provide an estimate of the degree of convergence/divergence of the panel on that particular question. The results of the data analysis were provided to the panelists for their review and confirmation.

## Results

The synthesized panelist recommendations for each A4R condition (and within this, each feature of the LEM) are listed in Tables 1 to 4 and are described below.

### A4R Condition Relevance

#### Features Related to Participation in Decision-Making

Regarding the feature *Facilitating participation in the committee of decision-makers that represent diverse perspectives*, panelists raised the question of who is entitled to participate and highlighted the need for a clear definition of the roles of each stakeholder as well as extent and form of participation (Table 1).

The general view was that, while legitimacy requires involvement of all stakeholders, the final decision (in a primarily publicly funded health system) rests within the elected decision maker (ie, governmental authorities). The reflective multicriteria approach was specifically recommended to facilitate participation, particularly for RD decision-making, where participation of patients was seen as a critical element. To promote understanding and ensure that all voices are heard, it was advised to provide well synthesized data and strive to reduce “charisma issues” in the committee. Representative participation during the process of evidence generation—ie, not only in evidence appraisal—was also recommended.

#### Features Related to Decision Criteria

Panelists offered diverse views on who should be involved in *Criteria selection*, with a trend towards opening the process to the wider public and engaging all stakeholders (Table 1). There was also an emphasis on the importance of reflection about how the criteria can be rooted in the legal/constitutional framework, goals of healthcare, social norms and overall population values and preferences. Diverse views were expressed regarding the consistency of decision criteria: some panelists stressed that consistency is a pre-requisite of accountability and legitimacy (and there should not be major differences between rare and more common diseases), others advocated for a more tailor-made, flexible approach to criteria selection, especially with respect to RDs and RTs.

Among decision criteria related to the *Effect of intervention*, there was consensus on the importance of *Comparative effectiveness*, which was viewed as the cornerstone of the decision and directly rooted in the goal of healthcare (Table 1). Definition of a common health gain measure across diseases was raised as a general challenge. Additional challenges, specifically with respect to RD interventions, included comparator selection in therapeutic areas where the standard of care is palliation only, and the use of intermediate outcomes. These factors may, according to some but not all panelists, require adaption of assessment methods to RD specificities. Panelists agreed that *Patient-perceived health/patient-reported outcomes* (PRO) should always be considered; however, it was recommended that the same documentation standards should apply as with clinical endpoints. Panelists confirmed the value of both generic and disease-specific PRO measures. With respect to RDs, however, they highlighted the difficulty of validating disease-specific PRO instruments in the RD setting. While the majority (6/7) regarded *Safety/tolerability* as an important aspect of the coverage decision, one view was that this belongs to the realm of regulatory decision-making. Panelists also noted the lack of safety data at the time of the reimbursement decision. In the RD and RT settings, pharmacovigilance was deemed of limited usefulness. Four of the 7 panelists thought that the criterion *Type of benefit* should also be considered because patients may view interventions differently depending on the type of benefit they provide and also to capture collective benefits from prevention. However, panelists also stressed the inherent difficulties in ranking different types of benefits (eg, life extension versus quality of life improvement). Also, they noted, specifically with respect to RTs and RDs, that the type of benefit may not be known at the time of the decision. Non-generic criteria, specific to an intervention (eg, disease-specific outcomes) should be clearly defined and ideally harmonized across assessments of different treatments for the same RD or RT. The latter, however, is not always feasible, particularly for interventions targeting RDs and RTs, which may have very specific outcome measures that may be tailored to the mode of delivery and the specific impact of the intervention.

Regarding the *Disease severity and unmet needs* domain, 4 panelists recommended considering the *Availability of alternatives (unmet needs)*, whereas one did not see this as a robust decision criterion (Table 1). Panelists raised the challenge of identifying the appropriate alternatives (or comparators), particularly for RD therapies. Consideration of *Disease severity* was also recommended by a majority of panelists (5/7), because it was perceived to raise awareness of the goal of healthcare and reflected broadly shared social values. However, panelists noted that proper consideration of *Disease severity* required a metric or ranking of conditions, which would need to be defined collectively within a society.

The *Economics* domain contained five criteria: *Cost (price) of intervention*; *Consequences of intervention for other medical costs*; *Consequences of intervention for non-medical costs*; *Budget impact, affordability and opportunity costs*; and *incremental cost-effectiveness ratio (ICER)* (Table 1). All of these aspects were recommended to be considered by a majority of panelists, with *Budget impact, affordability and opportunity costs* gaining the greatest support overall (6/7). Consideration of *Cost (price) of intervention* was deemed essential (5/7), as decision-makers aim to maintain health system sustainability and use resources based on the value provided, while taking opportunity costs into account. Panelists highlighted the high costs of RD and RT therapies and the ethical challenge that these “personalized medicines” pose to societies that strive to uphold the principle of solidarity. Panelists debated how to justify these high costs, whether or not they lead to significant budget impacts. One view was that there could be special considerations regarding the cost of RD interventions, related to their type and underlying economics, but these would need to be verified by further empirical studies. In this context, it was also noted that societies may place a value on not abandoning patients who suffer from rare, high-cost diseases, an element that could be integrated into value assessment, eg, through ‘social cost value analysis.’ One panelist also suggested that healthcare program costs (ie, budget impact) rather than the cost per patient would be the more appropriate approach to consider opportunity costs in the RD setting.

Panelists tended to agree that considering the *Consequences of intervention for other medical costs* was logical (ie, “reflects general principles of health economics”) (5/7), but with respect to RDs and RTs it was noted with that these costs are often negligible compared to the costs of the therapy itself, except possibly for some highly innovative interventions that are able to obviate other therapies (Table 1). Consideration of *Consequences of intervention for non-medical costs* was viewed to be relevant from the perspective of patients and their families and important for diseases with high disability burden (4/7). However, an alternative view was that it may not be appropriate to consider costs that are not covered by the payer who is making the coverage decision.

Consideration of the *ICER* was found to be useful to inform decisions by a majority (4/7), as it related added benefits to added costs (Table 1). Although cost-effectiveness analysis was deemed applicable for comparing interventions that target the same disease, the validity of setting universal ICER thresholds to inform allocative efficiency/opportunity cost decisions across disease areas was challenged by several panelists. In this context, it was recommended to avoid using the cost-per-QALY ratio for treatments that have no mortality impact, and rather relate costs to specific outcomes (eg, events), which are more meaningful to decision-makers. One panelist pointed out that the use of the ICER as a criterion within an MCDA framework that also contained effectiveness and cost criteria should be avoided as it would amount to double-counting. The use of the ICER in the realm of RDs and RTs was generally viewed as problematic due to methodological difficulties (ie, small sample size) and the fundamental challenge of universal ICER thresholds (as mentioned above). The caveat that the ICER threshold may be used to justify high prices (ie, to raise prices to just below the ICER ceiling) was also raised, specifically in relation to RD/RT interventions.

Among criteria of the domain *Ethical, social and legal aspects*, considering the *Feasibility of implementing intervention* was deemed essential by most panelists (6/7) in order to ensure that potential benefits are realized in clinical practice (Table 1). This was found to have particular importance for RD therapies, eg, because some of them need to be delivered in tertiary referral centers. However, with respect to *Innovativeness*, the majority view (5/7) was that it should not be considered, as all meaningful aspects of innovation (‘making a difference’) are already covered by other criteria. A dissenting view was that innovativeness should, in principle, be considered in relation to dynamic efficiency, ie, the benefits gained over the longer term through investment in innovation. Panelists also offered diverse views on *Rarity/Size of affected population* and using rarity for *Prioritizing populations*. One view was that rarity should not be considered in isolation but in relation with other aspects, such as disease severity and complexity of care. Some raised also the point that the concept of rarity may be misused to justify higher prices. Others expressed that rarity is often considered distinctively in practice and called for an MCDA-based approach to evaluating RD interventions. A further view was that if RDs and RTs are indeed societal priorities, then dedicated sources of funding could be established. With respect to *Prioritized populations*, in general, panelists recommended that prioritizations should be aligned with the values of society through broader public engagement and should be incorporated into evaluations explicitly and systematically. *Political, historical and cultural considerations* can in practice be important, but should, if included in a decision-making framework, be considered in a qualitative approach.

A majority of panelists (5/7) recommended considering the *Degree of uncertainty related to evidence (quality of evidence*) (Table 1). Specific recommendations included to distinguish effect size from its (un-)certainty and to treat quality of evidence (ie, its relevance and validity) as a separate notion from ‘uncertainty.’ With respect to RDs, one view was that, because of smaller samples sizes, less stringent data quality requirements could be used; others, however, contended that uncertainty can only be accepted provisionally and needs to be addressed by generating reliable (real-world) evidence effectiveness, eg, in the context of conditional access schemes.

#### Features Related to Evidence

With respect to *Considering different types of evidence*, several panelists expressed the view that both scientific and colloquial evidence (eg, expert evidence) is needed, particularly with respect to RTs and RDs, but with justification and related to the parameter in question (Table 1). However, there was also the caveat that “allowing anything is likely to obscure rather than inform.” *Selection of evidence* should be driven by scientific robustness and also by its relevance to the question it is supposed to address. Recommendations on how to *Present evidence* to enhance and support deliberation included presenting synthesized evidence for each decision criterion in a ‘by-criterion report’ or to use a ‘systematic review style.’ Panelists generally stressed the need to be transparent about the strengths, weaknesses and gaps of the evidence.

#### Features Related to Deliberation

Regarding *Balancing the values at stake*, several panelists stressed the need to ensure that the diversity of values held by society is included in the decision-making committee’s deliberation, although the role of elected government representatives was also highlighted (Table 1). Explicitly assigning weights to these values could help inform and structure the deliberation; however, weighting should aim to support, not replace, the collective thinking and learning process, which must arrive at a compromise in balancing the diversity of values. With respect to the feature *Assessing the performance of the intervention*, panelists commented that effectiveness should be assessed based on real-world impact and performed by HTA experts using a methodology formerly endorsed by the committee, or alternatively, by the members of the committee themselves with participation of diverse stakeholders. With respect to *Including individual interpretations to reach a group equilibrium in formulating a decision*, panelists recommended to provide ample room for reflection in order to foster openness and discourage defensiveness. *Decision rules* are needed for the sake of transparency; however, they should be grounded “in deep reflection on priority setting and the goals of the health system and on opportunity costs” and not be used in a rigid algorithmic fashion. With respect to RD and RTs, one panelist stated that firmly sticking to the ICER threshold may prove legally and politically infeasible.

### A4R Condition 2: Publicity

Three features were defined to operationalize the publicity condition: *Transparency of criteria, evidence and approaches used to consider them*, *Understandability of reasoning behind the decision*, and *Clarity of values underlying the decision* (Table 2). In general, panelists recommended transparency about the evidence and methods used in the assessment as well as transparency about the values that underlie the decision. Specifically, it was recommended to make explicit the value judgments used in the process of generating and selecting evidence, to communicate honestly and understandably to the public (including the uncertainties involved in the decision), and to clarify the relative weights of the decision criteria used. Additional attention should be paid to justifying endpoint selection for RD and RT interventions. Furthermore, one panelist stressed the need to further develop and professionalize the art of documenting and public communication about the decision, particularly in view of difficult decisions for RDs and RTs.

### A4R Condition 3: Appeal and Revision

The third A4R condition was operationalized through two features: *Handling of disagreements from stakeholders*, thus addressing appeals, and *Handling new evidence or new context*, addressing revisions (Table 3).

With respect to *Handling of disagreements from stakeholders,* panelists recommended involvement of diverse stakeholders in the process as well as a high level of transparency and publicity, which includes explicit decision rules, public debates, and providing space for dissenting voices to be heard, such as by publishing minority reports (ie, separate report prepared by a group representing a numerical minority of a committee) (Table 3). With respect to *Handling new evidence or new context*, recommendations included establishing clear rules regarding reviews, which should include a definition of what would qualify as ‘new evidence,’ and also allowing the considering new pertinent evidence in a timely fashion. For RDs and RTs specifically, continuous evidence generation and documentation through coverage with evidence development (CED) and performance-based contracting were recommended.

### A4R Condition 4: Enforcement (or Implementation)

To ensure that all relevant criteria and evidence are considered in a deliberative process that is inclusive of diverse perspectives (enforcement of Condition 1 – Relevance), one recommendation was to implement external and independent quality reviews of final deliberations (Table 4). Another recommendation was to ensure representative inclusion of different perspectives (ie, stakeholders, different scientific disciplines), which would be particularly well suited for deliberations regarding RDs and RTs (Table 4). A third recommendation was to create conditions in the committee that would promote deliberation and collective learning (eg, facilitated discussion by a skilled, independent moderator).

With respect to ensuring publicity of the decision rationales, ie, enforcement of Condition 2 – Publicity, visualization tools were mentioned as one means for ensuring that the reasons behind decisions are understandable and transparent and the values on which they are based are clear (Table 4). This includes the role and influence of MCDA criteria weights. Panelists again stressed the need for promoting a culture of collective learning among diverse stakeholders.

To ensure revisability of the decision in light of new evidence or arguments (enforcement of Condition 2 – Appeal and Revision), panelists recommended making re-assessments an integral part of the HTA process and highlighted the importance of proper documentation of decision rationales to support re-assessments (Table 4). Furthermore, the objective and timing of a planned re-evaluation should be clearly defined, so that relevant evidence can be planned for and generated (eg, in the context of CED) to address the pressing questions and evidence gaps. A final recommendation was to further develop HTA methodology by conducting conceptual and empirical research on multicriteria decision-making approaches. These recommendations were general but, according to one panelist, would primarily apply to RDs and RTs.

## Discussion

In this study, we reflected on and collected recommendations on the features that would characterize a legitimate and fair healthcare coverage decision process, using A4R as a conceptual framework.

A key recommendation for meeting the Relevance condition was to ground the criteria for healthcare coverage decisions in the legal/constitutional framework and normative (ethical) foundations. This requires reflection on the fundamental goals of healthcare, in the context of a country’s constitution, and how to transform these into operationalizable decision criteria.^5,31^ Decision criteria also need to be informed by a society’s values and preferences, which involves seeking to incorporate the priorities of citizens in decision-making (eg, through social cost value analysis), an area where there is a need for more robust evidence development.^61^ In this study, among a list of 17 generic decision criteria, 13 were recommended by more than half of the panelists, highlighting, as noted in previous research,^62,63^ the wide range of factors that could be considered relevant to coverage decision-making. For RDs, specifically, a broad perspective on value has been recommended^64,65^ and the use of a range of scientific and value judgements have been documented in actual coverage decision-making practice.^66^

Although the majority of the panelists supported using some form of cost-effectiveness analysis to inform decision-making, the use of the cost-per-QALY ratio alone to allocate resources across the health system was challenged on methodological and ethical grounds, especially for interventions targeting RDs. Indeed, it has been previously claimed that failing to incorporate diverse ethical positions (eg, the rule of rescue, the rights-based approach, distributional justice/fairness considerations), this approach, which aims at maximizing the sum of QALYs across the population, may lead to prioritization decisions that are inconsistent with a society’s moral values.^29,48,67^ Cost-effectiveness ratios also do not provide information on affordability as the public provision of cost-effective interventions, may prove unaffordable for a health system.^68^ In addition, it has been argued on theoretical grounds that the cost-per-QALY threshold approach may fail to maximize health gain for a given amount of resources.^69^ Furthermore, therapies targeting RDs, although their financial impact was shown to be and remain limited,^70^ often fail to meet cost-per-QALY thresholds,^71^ which may be viewed as disadvantaging patients with these conditions. This failure has generally been attributed to fixed R&D expenditures coupled with a small market size, which necessitate high unit costs, and the difficulty of generating high-quality comparative effectiveness evidence due the rarity of the condition.^48^ While the validity of these arguments must be further examined in empirical research,^72^ panelists tended to agree that rarity raises issues—related to unmet needs, constraints in evidence generation, and possibly in pricing (ie, return on investment from a smaller market)—which may warrant special consideration. However, they also noted that rarity should not be considered in isolation but in relation to other aspects such as disease severity, patients’ age, and budget impact. Indeed, surveys of the general population^73-77^ or medical doctors^78^ do not support rareness *per se* as a prioritization criterion. Nevertheless, such surveys do frequently demonstrate agreement with the rights-based argument^75^ as well as a strong concern for fairness,^73^ and express support for prioritizing severe diseases that have high unmet needs,^73,77^ attributes that are characteristic of many rare conditions.^79^

Another key theme emerging from the recommendations was the importance of stakeholder involvement for legitimacy within the A4R framework. Abelson and colleagues provide a useful distinction within the term ‘stakeholder’ between the ‘public’ (ie, citizens who can broadly represent social values), ‘patients’ (ie, those who have experience with a specific technology or condition), and other stakeholders (ie, those with an organized interest in a technology or condition, such as providers, advocacy groups or industry).^80^ Stakeholder involvement in HTA can occur at different levels: communication, which means receiving information on the assessment and its outcomes; consultation, which involves providing perspectives, experiences or preferences to inform the assessment; and participation, which means collaborating as partners in the assessment itself.^80-82^ In RDs, specifically, the crucial role of patients at all stages of evidence development has increasingly been recognized,^83-85^ resulting in calls for their active involvement in the HTA process as well.^83^ This was echoed in the panelists’ comments (“RD patients are likely to be the best experts in their disease”) and recommendations. The reflective multicriteria approach was specifically recommended to facilitate incorporation of diverse stakeholder perspectives, particularly that of patients in the RD and RT context. Previously, multicriteria methods were shown to be applicable for incorporating patients’ perspectives to inform priority setting^86^ and for value appraisal in the RD context.^47,87,88^ Participation of stakeholders with diverse perspectives serves to ensure that all potentially relevant reasons are examined in decision-making and to allow collective learning to take place during the deliberation.^8,14^ This form of participation can potentially increase public trust in the decision and reduce the need for appeals. With respect to criteria selection, recommendations included conducting public deliberations and broad consultations to select and validate decision criteria, echoing the call for HTA agencies to “subject their decision-making criteria to public scrutiny by means of a democratic process.”^14^ Clearly, the specific purpose, type of stakeholder and level of public engagement (eg, consultation vs some form of participation) need to be clearly defined and the various models, proposed or implemented, further tested and evaluated in the legal and social context of specific health systems^80,83,89^ to ensure that these are effective and appropriate in enhancing the legitimacy of the decision-making process.

As clearly recognized and explicitly expressed in the panelists’ recommendations, diverse stakeholders may justifiably disagree on how to balance different, and often conflicting, decision goals. Therefore, creating conditions that foster deliberation is integral to legitimate decision-making. Deliberation is a form of learning that cannot be replaced through quantitative methods or the majority vote.^8,39,41^ It involves understanding the health intervention and one’s own values as well as sharing personal definitions, judgments and values (ie, ‘interpretive frames’) with others to question assumptions and engage in shared ethical reasoning.^8,14^ Reflective multicriteria approaches can support this learning by providing a framework to structure the available evidence relevant to each decision criterion and to allow the explicit expression of values as separate from scientific judgments.^5,14^ Additional panelists’ recommendations to support deliberation included:

- Providing good-quality synthetized information before the committee meeting;
- Having a skilled, independent moderator who is able to foster mutual learning, prevent defensiveness (“changing one’s mind should not be a sign of weakness”), and reduce the risk of dominant committee members from adversely controlling the course of the deliberation (ie, “reduce charisma issues”);
- Balancing values discursively; assigning numerical weights to decision criteria should inform and structure deliberations but not replace them;
- If *consensus* cannot initially be reached, providing additional time for reflection, or otherwise a practical *compromise* in order to meet the need for making decisions within a short timeframe.^90^

Panelists also recommended being fully transparent about the weaknesses and uncertainties related to a decision (in addition to the methods employed and the underlying reasons) and as well as giving room to dissenting voices in order to foster an honest public debate, while creating conditions that promote the free exchange of ideas (eg, Chatham House Rule). In this regard, the art of effective and accurate public communication should be further developed, eg, through visualization tools. An additional recommendation was to reveal all value judgments used in the process of generating and selecting evidence. Indeed, previous research has drawn attention to the many and diverse types of value judgments that are implicit in the HTA process—including, for example, in selecting endpoints, defining thresholds for meaningful benefit, or performing specific types of economic analyses—and recommended making these explicit to increase accountability and provide stakeholders the opportunity to fully participate in the discussion.^91^ Although a subject of debate,^39,41^ the extent and form of ethical analysis (ie, which of the many implied value judgments should be revealed and addressed) will need to be determined by what is relevant to the decision at hand,^40^ a question that could benefit from a structured multicriteria approach to evidence selection, synthesis and documentation.

Ensuring that the relevance condition of A4R is met implies revisability of the decision in light of new evidence or new arguments. Regarding this aspect, panelists’ recommendations included CED and well-defined evidence needs and timelines for re-evaluation. These recommendations are echoed by a recent EURORDIS position statement that advocates for a rapid effectiveness assessment, which, while providing provisional access, would define a research question for targeted evidence generation to inform a full evaluation at a pre-specified point of time.^65^

## Limitations

This work reports a reflection on a wide range of features of on how best to meet the A4R conditions for legitimacy in coverage decision-making processes, in general, and in the context of RD and RTs, specifically. This was an initial exploratory study; as this, there was, by design, no deliberate attempt to reach a consensus during the panel session, which may be seen as one limitation of this study. Thus, some recommendations may appear, at least on the surface, contradictory, because they were rooted in diverse often incommensurable considerations. Disagreements might have been resolved in the group given more time for reflection, clarification and communication. The study involved only a relatively small group of panelists who were not intended to be representative of a wider HTA community, a particular jurisdiction, institution or approach. Panelists reflected on the questions posed and exchanged their insights based on their experience and expertise. A larger group of panelists may have provided a more diverse set of views and recommendations or, alternatively, may have revealed some areas of thematic convergence. The discussion of the panelists was supported by the LEM, which is not a coverage decision-making framework in itself, but a platform to elicit and structure reflection on the wide range of aspects that could operationalize the A4R conditions within the HTA context. While this study focused on HTA and health technology coverage decision-making, the A4R framework itself is meant to be applicable to limit-setting decisions in healthcare in general and some of the present findings may be useful for other applications within the health sector. Overlaps between different LEM features could have made this approach somehow cognitively demanding for the panelists. However, this is partly inevitable and reflects the interrelatedness of aspects in an actual decision-making setting. Conversely, the LEM may lack features that might be important. Not to be seen as a static tool, it can be further elaborated by the research community to foster the debate on how to address legitimacy requirements in decision-making.

## Conclusion

Moving coverage decision-making processes towards enhanced A4R is a continuous effort that involves designing and refining approaches to support participation and multi-stakeholder deliberation, enhancing transparency, and defining decision criteria that reflect normative and societal objectives.^5^ RDs and RTs are an important area in which to spearhead these efforts. Reflective multicriteria approaches can support this endeavor by allowing the explicit consideration of the wide range of criteria that stakeholders may find relevant for a specific decision problem; facilitating stakeholder involvement (a particularly critical element for patients in the RD context); and providing a means for appraising scientific evidence, expressing diverse value perspectives and making value judgments explicit, which can be shared in committee deliberations. Further conceptual and empirical development of multicriteria approaches is warranted to support their application in accountable and reasonable decision-making.

## Acknowledgements

This work was supported by Pfizer Inc., New York City, NY, USA. The funder reviewed the panel workshop manual and the manuscript and approved the manuscript submission.

## Ethical issues

This study did not use data collected from human subjects, ethics approval therefore was not required.

## Competing interests

This study was funded by Pfizer Inc. MW, DS, RC, and MB are employees of analytica Laser, which has received consulting fees from Pfizer Inc. for the conduct of the study and writing of the manuscript. CP was an employee of Pfizer Inc. at the time of the study. PA, BA, PK, LM, ASS, MS, and MT have no conflict of interest.

## Authors’ contributions

MW, DS, RC, MB, and CP contributed to workshop design, panelist recruitment and data analysis. MW drafted the manuscript and DS, RC, MB, and CP reviewed and revised it. The panelists, PA, BA, PK, LM, ASS, MS and MT, provided the data and reviewed and revised the manuscript. All authors approved the manuscript for submission.

## Authors’ affiliations

^1^Analytica Laser, Montreal, QC, Canada. ^2^Analytica Laser, London, UK. ^3^Analytica Laser, New York City, NY, USA. ^4^National Health Care Institute (ZIN), Diemen, The Netherlands. ^5^Pfizer Inc, New York City, NY, USA (retired). ^6^Liège University, Liège, Belgium. ^7^Center for Public Health Research, University of Milan-Bicocca, Milan, Italy. ^8^National School of Public Health IMIENS-UNED, Madrid, Spain. ^9^Department of Public Health, University of Alcalá, Alcalá de Henares, Spain. ^10^University of Leeds, Leeds, UK. ^11^Division of Health Economics, German Cancer Research Center (DKFZ), Heidelberg, Germany. ^12^University of Heidelberg, Heidelberg, Germany. ^13^ASST Niguarda and Regione Lombardia, Welfare Directorate, Milano, Italy.

## Supplementary files


Supplementary file 1 contanis the following appendices:
**Appendix 1.** Legitimacy Exploration Matrix
**Appendix 2.** Literature Search Strategy for Background Literature to Support the Development of the Legitimacy Exploration Matrix (LEM)
**Appendix 3.** Selection of Criteria for the Letitimacy Exploration Matrix (LEM) to Explore the Relavence Condition
**Appendix 4.** Overview of the Qualitative Analysis and Synthesis of Panelists Responses

## Key messages

**Implications for policy makers**

Using the accountability for reasonableness (A4R) as a conceptual framework, this study has explored how to move coverage decision-making processes towards enhanced A4R.

- A key recommendation for meeting the A4R Relevance condition was to ground the criteria for healthcare coverage decisions in the legal/ constitutional framework and normative (ethical) foundations.
- The importance of stakeholder involvement was highlighted and a reflective multicriteria-based approach was recommended to facilitate incorporation of diverse stakeholder perspectives, particularly those of patients in the rare disease (RD) context.
- Since diverse stakeholders may justifiably disagree on how to balance different, and often conflicting, decision goals, a key recommendation was to create conditions during committee meetings that foster deliberation.
- Additional recommendations included being fully transparent about the uncertainties surrounding a decision and, in that regard, further developing the art of effective and accurate public communication.

**Implications for public**

Making decisions on whether a new treatment should be covered by the health system is difficult, especially when it comes to therapies for rare diseases (RDs) and complex new types of treatments, such as gene therapy. In order to be legitimate, these decisions should be: (1) based on relevant reasons, (2) transparent with respect to all factors that were considered, (3) revisable in light of new evidence or arguments, and (4) there should be mechanisms to enforce these conditions. We discussed and explored systematically what can be recommended for designing decision-making processes that best align with each of these conditions with the goal of ensuring that healthcare coverage decisions are reasonable and fair.
