# Supplementary material for: Moving Towards Accountability for Reasonableness – A Systematic Exploration of the Features of Legitimate Healthcare Coverage Decision-Making Processes Using Rare Diseases and Regenerative Therapies as a Case Study
Source: Int J Health Policy Manag. 2019 May 8;8(7):424–43. doi: 10.15171/ijhpm.2019.24 (PMC6706971; doi:10.15171/ijhpm.2019.24)
Supplement: Supplementary file 1 — contanis the following appendices: Appendix 1. Legitimacy Exploration Matrix Appendix 2. Literature Search Strategy for Background Literature to Support the Development of the Legitimacy Exploration Matrix (LEM) Appendix 3. Selection of Criteria for the Letitimacy Exploration Matrix (LEM) to Explore the Relavence Condition Appendix 4. Overview of the Qualitative Analysis and Synthesis of Panelists Responses [file ijhpm-8-424-s001.pdf]

Supplementary file 1

Contents

1 APPENDIX 1: LEGITIMACY EXPLORATION MATRIX........ 2

1.1 A4R CONDITION 1: RELEVANCE 2

1.2 A4R CONDITION 2: PUBLICITY........ 9

1.3 A4R CONDITION 3: APPEAL & REVISION 10

1.4 A4R CONDITION 4: IMPLEMENTATION........ 11

2 APPENDIX 2: LITERATURE SEARCH STRATEGY FOR BACKGROUND LITERATURE TO SUPPORT THE DEVELOPMENT OF THE LEGITIMACY EXPLORATION MATRIX (LEM) 12

3 APPENDIX 3: SELECTION OF CRITERIA FOR THE LETITIMACY EXPLORATION MATRIX (LEM) TO EXPLORE THE RELAVENCE CONDITION 14

4 APPENDIX 4: OVERVIEW OF THE QUALITATIVE ANALYSIS AND SYNTHESIS OF PANELISTS RESPONSES 19

1 APPENDIX 1: LEGITIMACY EXPLORATION MATRIX

1.1 A4R Condition 1: RELEVANCE

FEATURE	WHAT DOES THE FEATURE COVER?	COST/QALY APPROACH – CASE	ALGORITHMIC MCDA APPROACH - CASE	REFLECTIVE MULTICRITERIA APPROACH - CASE	SPECIFIC POINTS RELATED TO RDRTs	WHAT COULD BE RECOMMENDED FOR THIS FEATURE?
Facilitating participation in the committee of decision-makers that represent diverse perspectives	How does the approach facilitate participation of diverse perspectives (e.g., patients, healthcare professionals, administrators, citizens)?	Participation of individuals representing diverse perspectives may be<br>• facilitated by the focus on one dominant criterion with fairly clear decision rules<br>• challenging due to complexity of the cost-per-QALY methodology	Participation of individuals representing diverse perspectives may be<br>• facilitated by transparency and simplicity of concepts considered (depends in how it is presented to the committee)<br>• challenging depending on the complexity of the MCDA methodology used (depends on the mental distance created by the method)	Participation of individuals representing diverse perspectives may be<br>• facilitated by transparency and simplicity of concepts considered (depends in how it is presented to the committee)<br>• challenging due to the non-conventional use of numbers that are representing reflection rather than data	RDs: Patient knowledge and input particularly important	What could be recommended to facilitate participation of diverse perspectives (e.g., patients, healthcare professionals, administrators, citizens)?<br><br>*Any specifics for RDRTs?*
**FEATURES RELATED TO CRITERIA**						
Criteria selection process	• Who selects the criteria?<br>• Based on which overall goal/values?<br>• Is there consistency of criteria across decisions?	• Institution<br>• Maximize welfare<br>• Consistent since focused on cost-per-QALY	• Stakeholder consultation under institution guidance<br>• None specified<br>• Criteria defined on a case-by-case basis; should be numerical contrast the alternatives compared	• Institution proposing and consultation to approve<br>• Aims to be inclusive of the fundamental goals of the healthcare system<br>• Consistent since derived from the goals which are stable	No specific points	• What could be recommended regarding who should select the criteria?<br><br>• What could be recommended regarding overall goal/values from which criteria are derived?<br><br>• Should there be consistency of criteria across decisions?<br><br>*Any specifics for RDRTs?*
GENERIC CRITERIA AND RATIONALES (i.e., criteria that are not specific for an intervention and therefore are used across decisions)						
Domain: Effect of intervention						
Comparative effectiveness	Is the comparative effectiveness of the proposed intervention considered and why?	Considered systematically, as an inherent part of the cost-per-QALY ratio	Considered systematically, not as a generic criterion but as disease-specific outcomes	Considered systematically, as the goal of meeting patient needs includes providing most effective treatments	• RTs and RDs: various challenges to conducting conventional RCTs<br>• RTs: Potential for large efficacy gains versus conventional treatment but uncertainty on durability<br>• RDs: magnitude of health gain difficult to estimate due to small trials	Should the comparative effectiveness of the proposed intervention be considered and why?<br><br>*Any specifics for RDRTs?*

FEATURE	WHAT DOES THE FEATURE COVER?	COST/QALY APPROACH – CASE	ALGORITHMIC MCDA APPROACH - CASE	REFLECTIVE MULTICRITERIA APPROACH - CASE	SPECIFIC POINTS RELATED TO RDRTs	WHAT COULD BE RECOMMENDED FOR THIS FEATURE?
Type of benefit (e.g., curative treatment, preventive intervention)	Is the nature of the clinical benefit provided by the proposed intervention (e.g., symptom relief, life extension, cure, prevention) considered and why?	The QALY does not differentiate between types of benefits but this can be considered informally	Algorithmic MCDA does not include criteria that are conceptual (not numerical) but this can be considered informally	Considered systematically, as the goal of meeting patient needs includes providing most beneficial treatments	• Some RTs are designed to be potentially curative<br>• RD: often limited data on type of benefit due to lack of long-term studies	Should the nature of the clinical benefit provided by the proposed intervention (e.g., symptom relief, life extension, cure, prevention) considered and why?<br><br>*Any specifics for RDRTs?*
Safety/ tolerability	Is the safety and tolerability of the proposed intervention in relation to alternatives considered and why?	• Considered systematically within the cost-per-QALY ratio<br>• Some aspects of safety may not have a measurable impact on the QALY but are considered in addition to the ratio	Considered systematically, not as a generic criterion but as disease-specific outcomes	Considered systematically, as the goal of meeting patient needs includes providing safest treatments	• RTs: potential for significant harm (malignancy, infection)<br>• RDs: risks are difficult to estimate in small patient populations	Should safety and tolerability of the proposed intervention in relation to alternatives be considered and why?<br><br>*Any specifics for RDRTs?*
Patient-perceived health / patient-reported outcomes	Are patient-perceived health / patient-reported outcomes generated by the proposed intervention in relation to alternatives considered and why?	• Considered systematically within the cost-per-QALY ratio through the utility weight, which is an inherent component of the QALY that is used as measure of health<br>• Some aspects of patient-perceived health may not be included in the QALY but can be considered in addition to the ratio	Considered systematically, not as a generic criterion, but as disease-specific outcomes	Considered systematically, as the goal of meeting patient needs includes providing treatments that improve patient-perceived health / patient-reported outcomes	• RTs: if therapy is curative, important QoL benefits are expected<br>• RDs: Disease-specific QoL instruments may be lacking	Should patient-perceived health / patient-reported outcomes generated by the proposed intervention in relation to alternatives be considered and why?<br><br>*Any specifics for RDRTs?*
Domain: Disease Needs						
Availability of alternatives (unmet needs)	Are the availability of alternatives and their shortcomings in their safety/tolerability or in their ability to prevent, cure, or improve the targeted health condition or ameliorate patient-perceived health considered and why?	Considered in addition to the cost-per-QALY ratio	Algorithmic MCDA does not include criteria that are conceptual (not numerical) but this can be considered informally	Considered systematically, as the goal of serving the whole population equitably includes targeting therapeutic areas of greatest unmet needs (worst off)	• RTs: Conventional alternatives may be available but may have significant limitations with respect to type of benefit, efficacy, safety and QoL impact<br>• RDs: General lack of targeted therapies	Are the availability of alternatives and their shortcomings in their safety/tolerability or in their ability to prevent, cure, or improve the targeted health condition or ameliorate patient-perceived health considered and why?<br><br>*Any specifics for RDRTs?*

FEATURE	WHAT DOES THE FEATURE COVER?	COST/QALY APPROACH – CASE	ALGORITHMIC MCDA APPROACH - CASE	REFLECTIVE MULTICRITERIA APPROACH - CASE	SPECIFIC POINTS RELATED TO RDRTs	WHAT COULD BE RECOMMENDED FOR THIS FEATURE?
Disease severity	Is the severity of the targeted health condition with respect to mortality, morbidity, disability, impact on function and quality of life, and clinical course of patient and impact on caregiver considered and why?	Considered in addition to the cost-per-QALY ratio	Algorithmic MCDA does not include criteria that are conceptual (not numerical) but this can be considered informally	Considered systematically, as the goal of serving the whole population equitably includes targeting patients with greatest disease severity (worst off)	• RTs typically developed for rare, severe, or advanced conditions (but not necessarily)<br>• RDs are often severe, chronic, progressive and life-threatening, with a variety of medical, psychological and social impacts (but not necessarily)	Should the severity of the targeted health condition with respect to mortality, morbidity, disability, impact on function and quality of life, and clinical course be considered and why?<br><br>*Any specifics for RDRTs?*
Domain: Economics						
Cost (price) of intervention	Is the cost (price) of the proposed intervention (includes acquisition and administration) in relation to current alternatives considered and why?	Considered systematically, within the cost-per-QALY ratio, as part of the total incremental cost, with the goal of maximizing value for money for the healthcare system	Algorithmic MCDA model does not include costs. Cost is considered systematically in addition to the MCDA output.	Considered systematically, as the goal of upholding healthcare system sustainability includes selecting cost-efficient treatments (*definition of cost-efficient: performing in the best possible manner with the least waste of resources*)	• RTs have usually high up-front cost per patient but conventional comparators can also be cost-intensive<br>• RDs: Drug costs tend to increase with decreasing number of targeted patients (rarity of condition)	Should the cost (price) of the proposed intervention (includes acquisition and administration) in relation to current alternatives be considered and why?<br><br>*Any specifics for RDRTs?*
Consequences of intervention for other medical costs	Is the impact of the proposed intervention on other medical costs (apart from acquisition and administration costs) considered and why?	Considered systematically,, within the cost-per-QALY ratio, as part of the total incremental cost, with the goal of maximizing value for money for the healthcare system	Algorithmic MCDA model does not include costs. Cost is considered systematically in addition to the MCDA output.	Considered systematically, as the goal of upholding healthcare system sustainability includes selecting cost-efficient treatments	• RTs and RDs: economic consequences uncertain but savings in other medical costs possible if intervention is safe and prevents cost-intensive disease complications and/or slows progression	Should the impact of the proposed intervention on other medical costs (apart from interventions that are directly replaced) be considered and why?<br><br>*Any specifics for RDRTs?*
Consequences of intervention for non-medical costs	Is the impact of the proposed intervention on non-medical costs (e.g., lost productivity, care giver time, social services, disability costs, transportation) considered and why?	Can be considered through a separate cost-per-QALY analysis from the perspective of society (primary analysis is often from the perspective of the healthcare system)	Algorithmic MCDA model does not include costs. Cost is considered systematically in addition to the MCDA output.	Considered systematically, as the goal of upholding the healthcare system and its role in society includes selecting interventions that also preserve societal and individual resources	• RDs and RTs: If therapy changes disease course (e.g., delays disability) or provides a cure, important impacts on non-medical costs are expected<br>• RTs: Patients may need to be treated at specialized centers (cost and time implications for families)	Should the impact of the proposed intervention on non-medical costs (e.g., lost productivity, care giver time, social services, disability costs) be considered and why?<br><br>*Any specifics for RDRTs?*

FEATURE	WHAT DOES THE FEATURE COVER?	COST/QALY APPROACH – CASE	ALGORITHMIC MCDA APPROACH - CASE	REFLECTIVE MULTICRITERIA APPROACH - CASE	SPECIFIC POINTS RELATED TO RDRTs	WHAT COULD BE RECOMMENDED FOR THIS FEATURE?
Budget impact, affordability and opportunity costs	Are the budget impact, affordability and the opportunity cost of the proposed intervention considered and why?	• Budget impact considered in addition to the cost-per-QALY ratio<br>• (Of note: Minimization of opportunity costs is a core principle of the cost-per-QALY approach)	Algorithmic MCDA model does not include costs.<br>Cost is considered systematically in addition to the MCDA output (e.g., through budget impact analysis)	Considered systematically, on the basis of a budget impact analysis, as part of the goal of upholding healthcare system sustainability and potential displacement of resources	• RTs: Budget impact may be considerable if therapy targets non-rare condition (e.g., type I diabetes)<br>• RDs: budget impact usually low due to few patients	Should the budget impact, affordability and the opportunity cost of the proposed intervention be considered and why?<br><br>*Any specifics for RDRTs?*
Incremental cost-effectiveness ratio (ICER)	Is the incremental cost-effectiveness ratio of the proposed intervention considered and why?	Considered systematically, based on utility theory	Algorithmic MCDA model does not include costs.<br>Cost is considered systematically in addition to the MCDA output.	Not considered to avoid double counting, since the cost-effectiveness ratio represents a composite of other criteria (e.g., costs, comparative effectiveness, comparative PROs) which are considered individually as separate concepts	• RTs: Challenge to provide the type of effectiveness and economic evidence that is required for cost-effectiveness analyses<br>• RDs: ICERs are often very uncertain or fail to meet established thresholds	Should the incremental cost-effectiveness ratio of the proposed intervention be considered and why?<br><br>*Any specifics for RDRTs?*
Domain: Ethical, social and legal aspects						
Rarity / Size of affected population	Is the rarity of the condition and/or the size of the population targeted by the proposed intervention considered and why?	If rarity is established as a priority, it can be considered systematically in addition to the cost-per-QALY or through varying cost-per-QALY thresholds or QALY weights	If rarity is established as a priority, it can be considered informally in addition to the MCDA output	• Size of population: Considered systematically, as the goal of serving the whole population equitably includes serving as many individuals as possible<br>• Rarity: Considered if rare diseases deemed a priority on the basis that the goal of serving the whole population equitably may include prioritizing rare diseases (separate criterion) because of the high levels of unmet needs stemming from scientific and economic constraints linked to rarity	• Rarity as a prioritization criterion for coverage decision is a topic of public discussion	Should the rarity of the condition and/or the size of the population targeted by the proposed intervention be considered and why?<br><br>*Any specifics for RDRTs?*
Prioritized populations	Is prioritization of specific populations (e.g., vulnerable populations), as defined by policy decision-makers/ societies, considered and why?	If priorities are established, these can be considered systematically in addition to the cost-per-QALY or through varying cost-per-QALY thresholds or QALY weights	Algorithmic MCDA does not include criteria that are conceptual (not numerical) but if priorities are established, they can be considered informally in addition to the MCDA output	Considered if priorities are established on the basis that the goal of serving the whole population equitably may include prioritizing specific populations (e.g., the most vulnerable)	• Many RDRT therapies target children	Should prioritization of specific populations (e.g., vulnerable populations), as defined by policy decision-makers/ societies, be considered and why?<br><br>*Any specifics for RDRTs?*

FEATURE	WHAT DOES THE FEATURE COVER?	COST/QALY APPROACH – CASE	ALGORITHMIC MCDA APPROACH - CASE	REFLECTIVE MULTICRITERIA APPROACH - CASE	SPECIFIC POINTS RELATED TO RDRTs	WHAT COULD BE RECOMMENDED FOR THIS FEATURE?
Feasibility of implementing intervention	Is the capacity of the healthcare system to appropriately implement the proposed intervention with respect to infrastructure, organization, skills, legislation requirements etc. considered and why?	Considered systematically in addition to the cost-per-QALY ratio	Algorithmic MCDA does not include criteria that are conceptual (not numerical) but this can be considered informally	Considered systematically, as making decisions informed by context includes considering the feasibility of implementing the intervention	• RTs: Challenges related to scale up, manufacturing, portability and integration in current care configurations; complex legal and regulatory requirements<br>• RDs: lack of expertise, challenge of reaching the whole target population, availability of special care infrastructure	Should the capacity of the healthcare system to appropriately implement the proposed intervention with respect to infrastructure, organization, skills, legislation requirements etc.be considered and why?<br><br>*Any specifics for RDRTs?*
Political, historical and cultural considerations	Is the political, historical and cultural context considered and why?	Can be considered informally	Can be considered informally in addition to the MCDA output	Considered systematically, as making decisions informed by evidence and context includes considering the political, historical and cultural context	• RDs: high level of patient engagement and participation in research creates a specific culture	Should the political, historical and cultural context be considered and why?<br><br>*Any specifics for RDRTs?*
Innovativeness	Is the concept of innovation in its broad sense (e.g., innovative therapy, innovative program, innovative care organization) considered and why?	Considered systematically in addition to the cost-per-QALY ratio	Can be considered informally in addition to the MCDA output	Considered if innovativeness contributes to improving health and increasing knowledge in healthcare	• RTs can be considered highly innovative and some of them are continuously evolving<br>• RDs: Development of rare diseases therapies may broadly advance knowledge beyond a single disease area	Should the concept of innovation be considered and why?<br><br>*Any specifics for RDRTs?*
CRITERIA SPECIFIC TO AN INTERVENTION (not-generic)						
CRITERIA SPECIFIC TO AN INTERVENTION (not-generic)	How are intervention-specific criteria included and why? (e.g., outcomes)	Intervention-specific outcomes are integrated in the QALY measure	Intervention-specific outcomes included as criteria	Intervention-specific criteria (including outcomes) may be included as sub-criteria of the generic criteria	RDRTs: patient input to define specific outcomes most relevant to them	How should intervention-specific criteria be included and why? (e.g., outcomes)<br><br>*Any specifics for RDRTs?*
Uncertainty of evidence						
Degree of uncertainty related to evidence (quality of evidence)	Are the relevance, validity and completeness (e.g., publication bias) of the evidence supporting the proposed intervention as well as the degree of uncertainty related to this evidence considered and why?	Considered systematically through sensitivity analysis (e.g., probabilistic, deterministic, scenario analysis) to provide an estimate of the variability of the cost-per-QALY ratio<br><br>Underlying goal is to make decisions based on valid evidence	Considered systematically through sensitivity analysis (e.g., probabilistic, deterministic, scenario analysis) of the data inputs for the scoring functions to provide an estimate of the variability of the MCDA estimate<br><br>Underlying goal is to make decisions based on valid evidence	Considered systematically; relevance and validity of evidence can be considered through explicit criteria called "quality of evidence"; uncertainty in judgments on evidence can also be expressed through scoring ranges for all criteria<br><br>Underlying goal is to make decisions based on available knowledge	• RTs: durability of treatment effect and safety likely unknown<br>• RDs: rareness affects quality of all types of evidence (natural history, epidemiology, clinical data, economics);<br>• Validity of surrogate sometimes often unknown	Should the relevance and validity of the evidence supporting the proposed intervention as well as the degree of uncertainty related to this evidence be considered and why?<br><br>*Any specifics for RDRTs?*
**FEATURES RELATED TO EVIDENCE**						

FEATURE	WHAT DOES THE FEATURE COVER?	COST/QALY APPROACH – CASE	ALGORITHMIC MCDA APPROACH - CASE	REFLECTIVE MULTICRITERIA APPROACH - CASE	SPECIFIC POINTS RELATED TO RDRTs	WHAT COULD BE RECOMMENDED FOR THIS FEATURE?
Considering different types of evidence	What type of evidence is considered: scientific, colloquial (“*anything that that establishes a fact or gives reason for believing something*”[1]) imputed by logic, insights/ experiential?	Primarily scientific, sometimes colloquial scientific evidence when there is a lack of published scientific evidence (e.g., expert opinion) as a proxy of scientific evidence	Scientific evidence that can expressed numerically	Scientific, colloquial, imputed by logic, insights/experiential	• RDRTs: experiential evidence may be highly relevant	What type of evidence should be considered: scientific, colloquial (“anything that that establishes a fact or gives reason for believing something”[7]) imputed by logic, insights/experiential?<br><br>*Any specifics for RDRTs?*
Selection of evidence	What drives the selection of evidence to be included in the assessment?	• Selection driven by the aim of generating a relevant cost-per-QALY estimate<br>• Less systematic evidence selection for other criteria that are considered	Selection driven by the aim of providing useful numbers to operationalize the MCDA methodology	Selection driven by the aim of providing any relevant evidence to allow consideration of all relevant aspects for each criterion	No specific points	What should drive the selection of evidence to be included in the assessment?<br><br>*Any specifics for RDRTs?*
Presentation of evidence	How is the evidence presented to enhance clarity and support deliberation?	Report with a focus on cost-per-QALY methodology and results with key points in Executive Summary	Numerical evidence and criterion side-by side (“by-criterion report”)	Synthesis of different types of evidence and criteria side-by side (“by-criterion report”)	No specific points	How should the evidence be presented to enhance clarity and support deliberation?<br><br>*Any specifics for RDRTs?*
**FEATURES RELATED TO DELIBERATION**						
Balancing values at stake	• How are the values at stake balanced during the deliberation?<br>• Who balances the values at stake?	• Informally, with pre-set predominance of welfare maximization principle<br>• Committee members are balancing the output of the cost-per-QALY model with other informal aspects	• By assigning weights to criteria using an indirect method to elicit preferences (e.g., discrete choice experiment, swing weighting)<br>• MCDA weights for the MCDA model are pre-established by stakeholders/ methodologists before committee meeting<br>• Committee members are balancing the MCDA output with other informal aspects	• By assigning weights to criteria using a direct method to elicit values<br>• Combination of both (committee members are using their own weights and may consider weights from a consultation of stakeholders)	• RDRTs raise ethical dilemmas stemming from the critical tension between meeting individual patient needs, serving the whole population equitably, and ensuring sustainability of health systems raised by their development and appraisal	• How should the values at stake be balanced during the deliberation?<br><br>• Who should balance the values at stake?<br><br>*Any specifics for RDRTs?*

[1] Lomas J, Culyer T, McCutcheon C et al, 2005. Conceptualizing and combining evidence for health system guidance. Canadian Health Services Research Foundation. 2005

FEATURE	WHAT DOES THE FEATURE COVER?	COST/QALY APPROACH – CASE	ALGORITHMIC MCDA APPROACH - CASE	REFLECTIVE MULTICRITERIA APPROACH - CASE	SPECIFIC POINTS RELATED TO RDRTs	WHAT COULD BE RECOMMENDED FOR THIS FEATURE?
Assessing the performance of the intervention	• How is the performance of the intervention assessed?<br>• Who is assessing the performance?<br>*NOTE: In this context, performance is defined as how good an intervention is in regard to a specific decision criterion (e.g., highly efficacious=high performance with regard to efficacy)*	• Performance is predominantly assessed with the cost-per-QALY output and less formally for the other criteria considered<br>• Committee members are assessing the cost-per-QALY output along with other informal aspects	• Performance is measured using a scoring function which has been defined by stakeholders and methodologists<br>• Committee is presented with output of MCDA model established by stakeholders and methodologists before committee meeting<br>• Committee members are assessing the MCDA output along with other informal aspects	• Performance is measured with a scoring method capturing judgements on all types of evidence<br>• Committee members interpret the evidence which is presented to them for each criterion and assign scores that express their judgement on performance	• No specific points	• How should the performance of the intervention be assessed?<br>• Who should be assessing the performance?<br>*Any specifics for RDRTs?*
Including individual interpretations to reach a group equilibrium in formulating a decision	How are individual interpretations included/shared to reach a group equilibrium in formulating a decision? (e.g., consensus, vote)	• Open deliberation with committee members leading to decision<br>• Vote when consensus cannot be reached	• Based on stakeholders' weights and scores derived from the scoring function to create a stakeholder output, which is checked by committee members for face validity<br>• Vote when consensus cannot be reached	• Based on a visual summary of the committee members' inputs (representing the group reflection), which are derived from quantitative and qualitative inputs of each member, and which is checked by the committee for face validity<br>• Vote when consensus cannot be reached	No specific points	How should individual interpretations be included/shared to reach a group equilibrium in formulating a decision? (e.g., consensus, vote)<br>*Any specifics for RDRTs?*
Decision rules and uncertainty	• Are there decision rules to guide the decision-making? What are they?<br>• How is uncertainty in decision-making handled?	• ICER threshold serves as decision rule<br>• By examining ICER variability through sensitivity analyses	• No general rules since criteria differ across assessments<br>• By exploring through face validity exercise the meaning of what is measured	• No strict rules but consistency of approach allows establishing rules over time in specific contexts<br>• Uncertainty on the validity of the judgments made is explored with face validity exercises	No specific points	• Should there be decision rules to guide the decision-making? What should they be?<br>• How should uncertainty in decision-making be handled?<br>*Any specifics for RDRTs?*

1.2 A4R Condition 2: PUBLICITY

FEATURE	WHAT DOES THE FEATURE COVER?	COST/QALY APPROACH – CASE	ALGORITHMIC MCDA APPROACH - CASE	REFLECTIVE MULTICRITERIA APPROACH - CASE	SPECIFIC POINTS RELATED TO RDRTs	WHAT COULD BE RECOMMENDED FOR THIS FEATURE?
Transparency of criteria and evidence considered and approaches used to consider them	• Is the evidence that was considered and methods to select and synthesize the evidence made public?<br>• Are the criteria and the deliberative approach to consider them made public?	• Yes<br>• Yes for the predominant criterion (cost-per-QALY); approach to consider other criteria is less clearly communicated	• Yes<br>• Yes for the criteria included in the MCDA model; approach to consider other criteria is less clearly communicated	• Yes<br>• Yes	No specific points	• Should the evidence that was considered and the methods to select and synthesize the evidence be made public?<br>• Should the criteria and the deliberative approach to consider them be made public?<br><br>*Any specifics for RDRTs?*
Understandability of reasoning behind decision	How does the approach facilitate making reasons leading to the decision explicit and understandable to stakeholders, including the public?	Making reasons leading to the decision explicit and understandable to stakeholders may be<br>• facilitated by the focus on one dominant criterion with fairly clear decision rules<br>• challenging due to complexity of the cost-per-QALY methodology and the reasoning behind integrating the other criteria	Making reasons leading to the decision explicit and understandable to stakeholders may be<br>• facilitated by transparency and simplicity of concepts considered<br>• challenging due to the complexity of the algorithmic transformation of the criteria in the MCDA output and the reasoning behind integrating the other criteria	Making reasons leading to the decision explicit and understandable to stakeholders may be is<br>• facilitated by transparency and simplicity of concepts considered as well as visualization of the reasoning leading to the decision<br>• challenging due to the non-conventional use of numbers that are representing reflection rather than data	No specific points	What could be recommended to facilitate making reasons leading to the decision explicit and understandable to stakeholders, including the public?<br><br>*Any specifics for RDRTs?*
Clarity of values underlying the decision	Are values underlying decisions stated? Is there a reference to the broader objectives and underlying mandate of the agency/institution/ healthcare system?	Welfare maximization is the underlying principle of the cost-per-QALY approach	Not inherent to the approach	Criteria represent the fundamental goals of healthcare and the way they were balanced represent the relative importance of the values underlying the decision	No specific points	Should values underlying decisions be stated? Should there be a reference to the broader objectives and underlying mandate of the agency/institution/healthcare system?<br><br>*Any specifics for RDRTs?*

1.3 A4R Condition 3: APPEAL & REVISION

FEATURE	WHAT DOES THE FEATURE COVER?	COST/QALY APPROACH – CASE	ALGORITHMIC MCDA APPROACH - CASE	REFLECTIVE MULTICRITERIA APPROACH - CASE	SPECIFIC POINTS RELATED TO RDRTs	WHAT COULD BE RECOMMENDED FOR THIS FEATURE?
Handling of potential disagreements from stakeholders	How does the approach facilitate consultation to collect feedback from stakeholders on interpretation of data, rationale for decision, values considered and/or reduce the need for appeal?	Public consultation may be<br>• facilitated by the focus on one dominant criterion with fairly clear decision rules<br>• challenging due to complexity of the cost-per-QALY methodology and the reasoning behind integrating the other criteria	Public consultation may be<br>• facilitated by transparency and simplicity of concepts considered<br>• challenging due to the complexity of the algorithmic transformation of the criteria in the MCDA output and the reasoning behind integrating the other criteria	Public consultation may be<br>• facilitated by transparency and simplicity of concepts considered as well as visualization of the reasoning leading to the decision<br>• challenging due to the non-conventional use of numbers that are representing reflection rather than data	No specific points	What could be recommended to facilitate consultation to collect feedback from stakeholders on interpretation of data, rationale for decision, values considered and/or reduce the need for appeal?<br><br>*Any specifics for RDRTs?*
Handling new evidence or new context	How does the approach facilitate considering new evidence or new context?	New evidence or context requires generating a new ICER	New evidence may require re-eliciting weights and re-defining scoring scales or creating new ones (new outcome)	New evidence requires revising judgments	• Evidence generation may be ongoing post-approval and (e.g., real-world studies, registries) maturity of evidence and decreased uncertainty likely to be gained over time	What could be recommended to facilitate considering new evidence or new context?<br><br>*Any specifics for RDRTs?*

1.4 A4R Condition 4: IMPLEMENTATION

FEATURE	WHAT DOES THE FEATURE COVER?	COST/QALY APPROACH – CASE	ALGORITHMIC MCDA APPROACH - CASE	REFLECTIVE MULTICRITERIA APPROACH - CASE	SPECIFIC POINTS RELATED TO RDRTs	WHAT COULD BE RECOMMENDED FOR THIS FEATURE?
Existence of means to enforce condition 1	Is there a process to ensure that all relevant criteria and evidence are considered in a deliberative process that is inclusive of diverse perspectives?	There is a process to ensure that the ICER and other criteria and relevant evidence are considered; the deliberative process does not guarantee that committee members' interpretive frames are integrated in the final decision	The algorithmic MCDA process ensures that the criteria of the MCDA model (selected on a case-by-case basis) and additional criteria and relevant evidence are considered.<br>The deliberative process does not guarantee that committee members' interpretive frames on all the criteria are integrated in the final decision	The reflective multicriteria process ensures that all criteria derived from fundamental goals and relevant evidence are considered and that committee members' interpretive frames are integrated in the final visual summary which leads to the decision	No specific points	What could be recommended to ensure that all relevant criteria and evidence are considered in a deliberative process that is inclusive of diverse perspectives?<br><br>*Any specifics for RDRTs?*
Existence of means to enforce condition 2	Is there a process to ensure publicity of the rationales of the decision, i.e., that the reasons behind decisions are understandable and the values on which they are based are clear?	There is a process to ensure that the rationales for the decision are made public. The reasons behind decisions are dominated by the ICER reflection and the welfare maximization is clearly conveyed as the key principle underlying the decision.	There is a process to ensure that the rationales for the decision are made public. The reasons behind decisions include the MCDA output and other criteria. The underlying values on which the decision is based is not clearly specified.	There is a process to ensure that the rationales for the decision are made public. The reasons behind decisions include the balancing of the fundamental goals of healthcare which are clearly conveyed as the principles underlying the decision.	No specific points	What could be recommended to ensure publicity of the rationales of the decision, i.e., that the reasons behind decisions are understandable and the values on which they are based are clear?<br><br>*Any specifics for RDRTs?*
Existence of means to enforce condition 3	Is there a process to ensure revisability of the decision in light of new evidence or arguments?	There is a process to ensure revisability of the decision in light of new evidence or arguments which requires generating a new ICER	There is a process to ensure revisability of the decision in light of new evidence or arguments which requires re-eliciting weights and re-defining scoring scales or creating new ones (new outcome)	There is a process to ensure revisability of the decision in light of new evidence or arguments which requires revising judgments	No specific points	What could be recommended to ensure revisability of the decision in light of new evidence or arguments?<br><br>*Any specifics for RDRTs?*

2 APPENDIX 2: LITERATURE SEARCH STRATEGY FOR BACKGROUND LITERATURE TO SUPPORT THE DEVELOPMENT OF THE LEGITIMACY EXPLORATION MATRIX (LEM)

The PubMed/Medline bibliographic database of life sciences and biomedical information were searched in April 2017 using the strategies below.

Search	Search terms	Number of items screened	Selection criteria
**1**	"accountability for reasonableness" OR A4R<br>Filters: none	101	• Discusses "accountability for reasonableness" and its application
**2**	(value AND (assess* OR apprais* OR framework)) OR "health technology assessment" OR HTA OR reimbursement OR cost* OR funding OR coverage OR hurdle OR priority OR prioritization OR prioritisation OR appraisal OR (resource AND allocation) OR economic*<br>AND<br>"rare disease" OR "rare diseases" OR "orphan disease" OR "orphan diseases" OR "rare disorder" OR "rare disorders" OR "rare diseases"[MeSH] OR "regenerative therapy" OR "regenerative medicine" OR "gene therapy" OR "stem cell therapy" OR "cell therapy"<br>Filters: Abstract available; Publication date from 2012/01/01	2853	• Discusses cost-per-QALY or multicriteria-based approaches to reimbursement decision-making that could be relevant to rare disease and regenerative therapies (RDRTs)<br>or<br>• Discusses issues that can potentially impact appraisal / decision-making for RDRTs
**3**	(multicriteria decision analysis OR multi-criteria decision analysis OR MCDA OR multi-criteria OR multicriteria OR analytical hierarchy process OR multiattribute utility analysis OR multi-attribute utility analysis OR MCDM OR EVIDEM) NOT (water OR chemical OR chemistry OR food)<br>OR<br>Value assessment framework<br>Filters: published in the last 10 years	2360	• Discusses multicriteria-based approaches to reimbursement decision-making that could be relevant to RDRTs<br>or<br>• Discusses issues that can potentially impact appraisal / decision-making for RDRTs
**4**	(cost-utility OR cost-effectiveness OR cost per QALY OR ICER OR cost/QALY)<br>AND<br>"rare disease" OR "rare diseases" OR "orphan disease" OR "orphan diseases" OR "rare disorder" OR "rare disorders" OR "rare diseases"[MeSH] OR "regenerative therapy" OR "regenerative medicine" OR "gene therapy" OR "stem cell therapy" OR "cell therapy"<br>Filters: none	338	• Discusses cost-per-QALY-based approaches to reimbursement decision-making that could be relevant to RDRTs<br>or<br>• Discusses issues that can potentially impact appraisal / decision-making for RDRTs

ICER: incremental cost-effectiveness ratio; QALY: quality-adjusted life-year; RDRT: rare disease and regenerative therapies;

Combined, these searches yielded a total of 5700 unique items, which were screened on a title and abstract basis. Of these, a total of 237 full-text journal articles were reviewed in full text.

Search strategy #3 was specifically utilized to systematically identify full-text articles that reported on (**inclusion criteria**):

1. Multicriteria-based approaches /frameworks for supporting healthcare system reimbursement or coverage decision-making ***and***
2. Were explicitly claimed by their developers to be applicable to decision-making on coverage of therapies for rare / orphan diseases

The following articles were excluded (**exclusion criteria**):

1. Framework developed for applications other than coverage decision-making, such as risk-benefit assessment or patient-level decision-making
2. Framework not specific to rare / orphan diseases
3. Framework more fully described elsewhere
4. Not MCDA (e.g., expansion or adaptation of cost-utility analysis)
5. Abstract only / no full-text
6. Review/analysis of current decision-making processes or published literature or survey

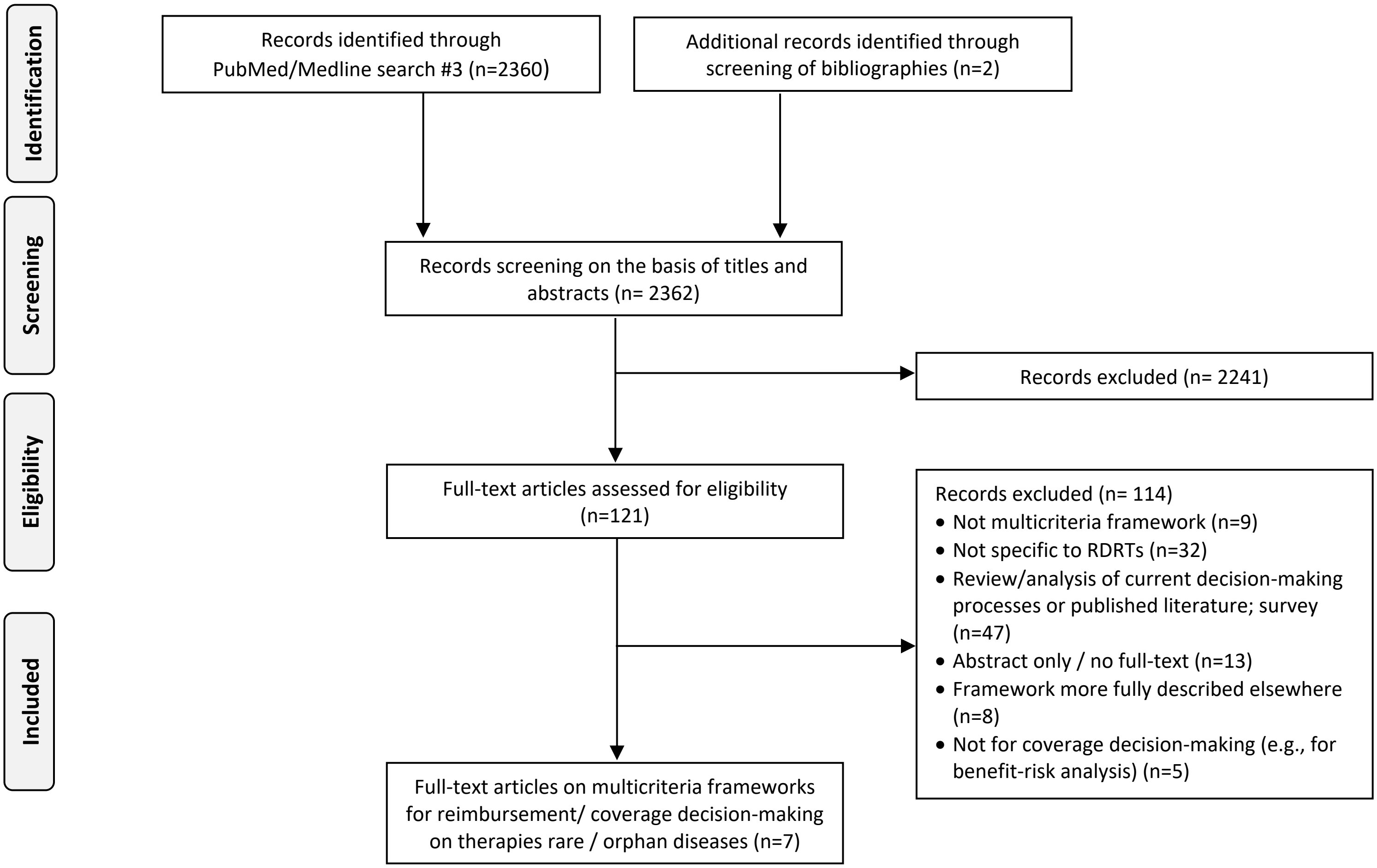

From these literature searches, a total of seven published multi-criteria frameworks were proposed to be responsive and applicable to appraisals of interventions targeting rare diseases were thus identified:

- Framework of the Working Group on Mechanism of Coordinated Access to Orphan Medicinal Products (WG on MoCA-OMP)[2]
- Hughes-Wilson et al, 2012[3]
- Sussex et al, 2013[4]
- Iskrov, et al, 2016[5]
- Kolasa et al 2016[6]
- Paulden et al 2015[7] and
- Wagner, et al 2016[8]

3 APPENDIX 3: SELECTION OF CRITERIA FOR THE LETITIMACY EXPLORATION MATRIX (LEM) TO EXPLORE THE RELAVENCE CONDITION

The table below compares and matches the decision criteria proposed in the seven identified multicriteria frameworks. As a rule, a criterion was included in the LEM if it was featured in at least two frameworks, unless the rationale offered for the proposed criterion was based on price justification rather than value (i.e., the criterion indicated a higher level of investment or a lower potential for revenue).

---

[2] European Commission. Process on Corporate Social Responsibility in the Field of Pharmaceuticals Platform on Access to Medicines in Europe Working Group on Mechanism of Coordinated Access to Orphan Medicinal Products (MoCA-OMP). Transparent value framework. 2014.

[3] Hughes-Wilson W, Palma A, Schuurman A, Simoens S. Paying for the Orphan Drug System: break or bend? Is it time for a new evaluation system for payers in Europe to take account of new rare disease treatments? Orphanet J Rare Dis 2012;7:74

[4] Sussex J, Rollet P, Garau M, Schmitt C. A pilot study of multicriteria decision analysis for valuing orphan medicines. Value Health 2013;16(8):1163-9.

[5] Iskrov G, Miteva-Katrandzhieva T, Stefanov R. Multi-Criteria Decision Analysis for Assessment and Appraisal of Orphan Drugs. Front Public Health 2016;4:214

[6] Kolasa K, Zwolinski KM, Kalo Z, Hermanowski T. Potential impact of the implementation of multiple-criteria decision analysis (MCDA) on the Polish pricing and reimbursement process of orphan drugs. Orphanet J Rare Dis 2016;11:23

[7] Paulden M, Stafinski T, Menon D, McCabe C. Value-based reimbursement decisions for orphan drugs: a scoping review and decision framework. Pharmacoeconomics 2014;33(3):255-69.

[8] Wagner M, Khoury H, Willet J, Rindress D, Goetghebeur M. Can the EVIDEM framework tackle issues raised by evaluating treatments for rare diseases: analysis of issues and policies, and context-specific adaptation. Pharmacoeconomics 2016;34(3):285-301.

Decision criteria comparison and matching across seven multi-criteria frameworks proposed to be applicable to rare disease coverage decision-making

	WG on MoCA-OMP[2]	Hughes-Wilson, 2012[3]	Sussex, 2013[4]	Iskrov, 2016[5]	Kolasa, 2016[6]	Paulden, 2015[7]	Wagner, 2016[8]
**CRITERIA INCLUDED IN THE LEGITIMACY EXPLORATION MATRIX**							
**Availability of alternatives (unmet needs)**	• Available alternatives/ unmet need	• Availability of alternatives (unmet needs)	• Availability of effective treatment options/ best supportive care in the absence of the new medicine (unmet needs)	• Alternatives (yes/no)	• Therapeutic alternative (unmet medical need)	• Availability of treatment alternatives	• Unmet needs
**Disease severity**	--	• Disease severity	• Disease survival prognosis with current standard of care (Disease severity – impact on survival)<br>• Disease morbidity and patient clinical disability with current standard of care (Disease severity – impact on morbidity and disability)<br>• Social impact of the disease on patients' and carer daily lives with current standard of care	• Disease severity	• Disease severity	• Severity (seriousness) of disease<br>• Extent to which the disease is life-threatening or chronically debilitating without treatment	• Disease severity (Impact on life-expectancy; Impact on morbidity; Impact in patient QoL; Impact on caregiver QoL)
**Comparative effectiveness**	• (Relative) effectiveness, degree of net benefit (clinical improvement, QoL, etc. vs side effects, societal impact, etc.) relative to alternatives<br>• Response rate (based on best available clinically relevant criteria)	--	• Evidence of treatment clinical efficacy and patient clinical outcome	• Clinical effectiveness	--	• Evidence of treatment efficacy or effectiveness	• Comparative effectiveness<br>○ Outcome 1<br>○ Outcome 2
**Type of benefit**	--	• Level of impact on condition/disease modification (type of clinical benefit)	--	• Type of health benefit (cure max, QoL min)<br>• Life-saving (yes/no)<br>• Prevention (yes/no)	--	• Magnitude of treatment benefit	• Type of therapeutic benefit<br>• Type of preventive benefit
**Safety / tolerability**	• (Relative) effectiveness, degree of net benefit (clinical improvement, QoL, etc. vs side effects, societal impact, etc.) relative to alternatives	--	• Treatment safety	• Safety	• Benefits from use of medicine (safety and adverse effects)	• Safety profile of treatment	• Comparative safety / tolerability (adverse events [AEs], serious AEs, fatal AEs, long-term safety, tolerability)

	WG on MoCA-OMP[2]	Hughes-Wilson, 2012[3]	Sussex, 2013[4]	Iskrov, 2016[5]	Kolasa, 2016[6]	Paulden, 2015[7]	Wagner, 2016[8]
**Patient-perceived health / patient-reported outcomes**	• (Relative) effectiveness, degree of net benefit (clinical improvement, QoL, etc. vs side effects, societal impact, etc.) relative to alternatives	--	• Social impact of the treatment on patient and carer daily lives	--	--	--	• Comparative patient-perceived health/ PROs (impact of intervention on HRQoL, autonomy, dignity, convenience /ease of use/mode of administration)
**Degree of certainty (quality of evidence)**	• Degree of certainty (documentation)	• Level of uncertainty of effectiveness	--	• Strength of evidence	• Scientific evidence for clinical efficiency (level of uncertainty)	--	• Quality of evidence<br>• Expert consensus/ clinical practice guidelines
**Cost (price) of treatment**	--	--	--	--	--	• Cost (price) of treatment	• Comparative cost consequences – cost of intervention
**Economic consequences of treatment on other medical costs**	--	--	--	--	--	• Societal impact of treatment	• Comparative cost consequences –Other medical costs to HC system<br>• Comparative cost consequences –Medical costs to patient
**Economic consequences of treatment on non-medical costs (e.g., productivity, social care costs)**	--	--	--	--	--	• Societal impact of treatment	• Comparative cost consequences – Patient/caregiver productivity<br>–Costs to wider social care system<br>–Non-medical costs to patient
**Budget impact, affordability and opportunity costs**	--	--	--	• Budget impact	• Budget impact (in €)	• Budget impact of treatment	• Affordability and opportunity costs (opportunity costs [forgone resources] for patient and for population)
**Cost-effectiveness**	--	--	--	• Cost-effectiveness (ICER)	• Cost effectiveness	--	--
**Rarity / Size of affected population**	--	• Rarity	--	--	• Disease rarity	• Prevalence (rarity) of disease<br>• Impact of disease upon the distribution of health in the population	• Population priorities - Rare diseases<br>• Size of affected population
**Prioritized populations (e.g., children)**	--	--	--	• Vulnerable groups (e.g., children)	--	--	• Population priorities - Other priorities
**Innovativeness**	--	--	• Treatment innovation, defined as the scientific advance of the new treatment together with contribution to patient outcome	--	• Advancement of technology	• Innovation profile of treatment	• Political, historical and cultural context (impact on innovation & research)

	WG on MoCA-OMP[2]	Hughes-Wilson, 2012[3]	Sussex, 2013[4]	Iskrov, 2016[5]	Kolasa, 2016[6]	Paulden, 2015[7]	Wagner, 2016[8]
**Feasibility of implementing intervention**	--	--	-	--	--	• Feasibility of diagnosing the disease<br>• Feasibility of providing treatment<br>• Identifiability of the beneficiaries of treatment	• System capacity and appropriate use (organizational [e.g., process, premises, equipment], skill, legislative and surveillance requirements; risk of inappropriate use; barriers to uptake; ability to reach the whole target population)
**Legal, political, historical and cultural considerations**	--	--	--	--	--	• Industrial and commercial policy considerations<br>• Socioeconomic policy objectives<br>• Legal considerations	• Political, historical and cultural context (political priorities and context; cultural acceptability; precedence [congruence with previous and future decisions]; impact on innovation & research; impact on partnership & collaboration among healthcare stakeholders)
**CRITERIA NOT INCLUDED IN THE LEGITIMACY EXPLORATION MATRIX**							
**Reason for exclusion**							
**Criterion can be used to justify higher price - Not a value element**	--	• Number of indications (rationale offered: a drug with a single indication has a lower potential for return on investment than a drug with multiple indications)	--	--	• Indication uniqueness (higher scores for drugs with unique indication)	-	--
	--	• Manufacturing complexity (rationale offered: manufacturing costs are higher for more complex therapies)	--	--	• Manufacturing technology complexity (higher scores for drugs with higher complexity)	--	-
	--	• Follow-up measures required (rationale offered: additional safety and efficacy studies post-approval are costly)	--	--	--	--	--
	--	• Level of research undertaken (rationale offered: research to generate data on natural history, cost burden etc. is costly)	--	--	-	--	--
**Cited by one framework only**	--	--	--	--	--	• Impact of treatment upon the distribution of health in the population	--

	WG on MoCA-OMP[2]	Hughes-Wilson, 2012[3]	Sussex, 2013[4]	Iskrov, 2016[5]	Kolasa, 2016[6]	Paulden, 2015[7]	Wagner, 2016[8]
	--	--	--	• Disease burden (sum of direct non-health-care costs, productivity loss, and early retirement costs)	--	--	--
	--	--	--	--	--	--	• Alignment with the mandate and scope of healthcare system
	--	--	-	--	--	--	• Alignment with the common goal rather than special interests
	--	--	--	--	--	--	• Environmental sustainability

4 APPENDIX 4: OVERVIEW OF THE QUALITATIVE ANALYSIS AND SYNTHESIS OF PANELISTS RESPONSES

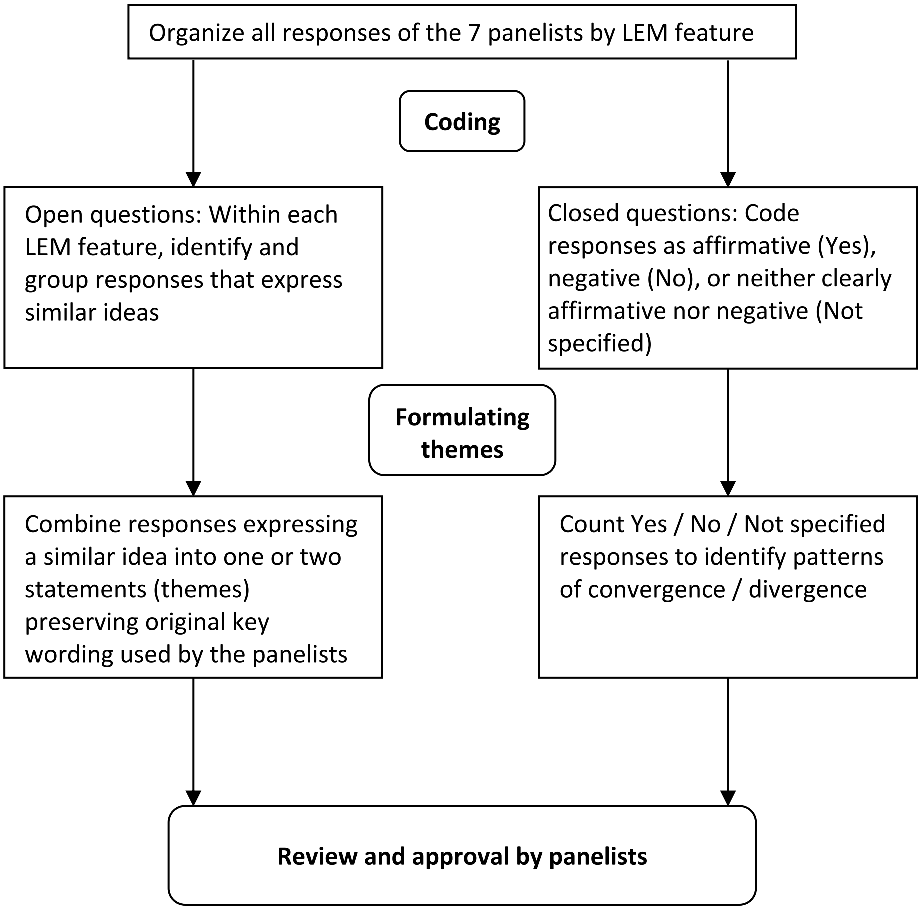

LEM: legitimacy exploration matrix
